# The (ab)use of food frequency questionnaire data in substitution modelling in nutritional epidemiology: a critique

**DOI:** 10.1038/s41430-026-01712-7

**Published:** 2026-02-22

**Authors:** Jimmy Chun Yu Louie, Jahar Bhowmik

**Affiliations:** 1 Discipline of Dietetics, Department of Allied Health, Swinburne University of Technology, Hawthorn, VIC Australia; 2 Department of Biomedical, Health and Exercise Sciences, School of Health Sciences, Swinburne University of Technology, Hawthorn, VIC Australia

**Keywords:** Epidemiology, Nutrition

## Abstract

**Background/Objectives:**

Food Frequency Questionnaires (FFQs) are widely used in nutritional epidemiology, particularly in substitution modelling to estimate health effects of dietary changes. This review examines validation practices in substitution modelling studies using FFQ-derived data published between 2018 and 2024.

**Subjects/Methods:**

A total of 100 studies from 21 countries were included. We assessed the presence and quality of validation data for FFQ variables used in substitution models, focusing on reported validation metrics and correspondence with reference methods.

**Results:**

Fifty-three percent of studies used unvalidated FFQ-derived variables in modelling. Among those providing validation data, correlation coefficients with reference methods ranged from 0.12 to 0.77 (median, Q1–Q3: 0.43, 0.30 to 0.50). Minimal or no documentation was found in 62% of studies. In some cases, deviations from reference values exceeded 450%. Studies using unvalidated inputs were frequently published in high-impact journals.

**Conclusions:**

The widespread use of unvalidated FFQ variables and the broad variability in validation quality raise concerns about the reliability of substitution modelling outcomes. Given the role of these studies in informing dietary guidelines, consistent validation protocols and improved reporting standards are urgently needed.

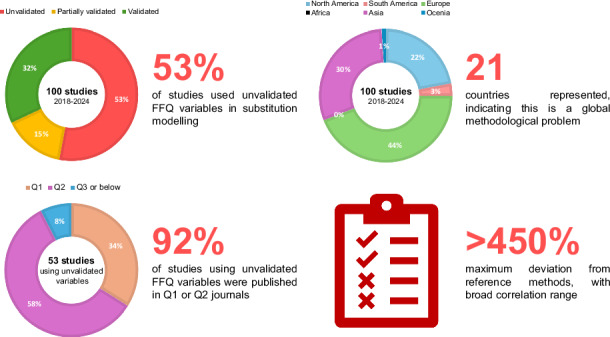

## Introduction

Understanding the relationship between dietary patterns and health outcomes represents one of the most challenging areas in modern epidemiological research. Unlike pharmaceutical trials or other medical investigations, where randomized controlled trials (RCTs) serve as the gold standard, nutrition research faces unique constraints [1–4]. Ethical considerations prevent randomising individuals to potentially harmful diets [5], while practical challenges include the difficulty of achieving adequate dietary adherence over the extended periods needed to demonstrate meaningful health impacts [5, 6].

Given these constraints, nutritional epidemiology has largely relied on observational studies, with Food Frequency Questionnaires (FFQs) becoming the predominant dietary assessment tool due to their practicality and cost-effectiveness in large populations [7, 8]. These questionnaires are designed to capture habitual dietary intake over extended periods, making them particularly suitable for longitudinal research examining diet-disease relationships [7].

Within observational nutrition research, substitution modelling has emerged as a key analytical approach for understanding how dietary modifications might influence health outcomes [9, 10]. Traditional diet-outcome analyses typically employ multivariable adjustment including multiple dietary factors even when focusing on a primary dietary component [11], but the analytical emphasis remains on individual associations rather than the quantified replacement effects that are central to substitution modelling [12]. Two common types of substitution models, the ‘*leave-one-out*’ and ‘*energy partitioning*’ models, have been widely utilised in research [9, 10]. These models have been thoroughly examined and their theoretical foundations, advantages and disadvantages are detailed in the literature [9].

However, the widespread use of FFQ data in substitution modelling raises serious methodological concerns. While FFQs have well-documented limitations affecting all types of dietary analyses, several aspects of substitution modelling make these limitations particularly problematic [7, 8, 13]. Most critically, substitution modelling requires accurate quantification of both the replaced and replacement foods, introducing compounded measurement error that may be less prominent in traditional analyses focusing on single dietary components. Despite these challenges, the extent to which unvalidated or poorly validated FFQ variables are used in substitution modelling remains unclear. We aim to examine this critical methodological issue by systematically evaluating validation practices in substitution modelling studies published in recent years.

## Methods

### Search strategy and study selection

We conducted targeted searches on 1st January 2025 in MEDLINE, PubMed and CINAHL databases to identify relevant studies published between January 2018 and December 2024. We chose 2018 as our starting point to capture recent practices in the field while ensuring a substantial sample size, representing current methodological approaches in nutritional epidemiology after several important methodological papers highlighting FFQ limitations [14–17]. The search strategy combined terms related to food frequency questionnaires and substitution modelling. The core search terms included: (‘food frequency questionnaire’ OR ‘FFQ’) AND (‘substitution’ OR ‘substitution model*’ OR ‘replacement’ OR ‘isocaloric substitution’). Hand searching of the reference lists of the identified studies was not performed. While this search strategy may not capture all studies using substitution analyses as secondary analyses, it provides a representative sample of studies explicitly focusing on substitution modelling.

**Fig. 1:**
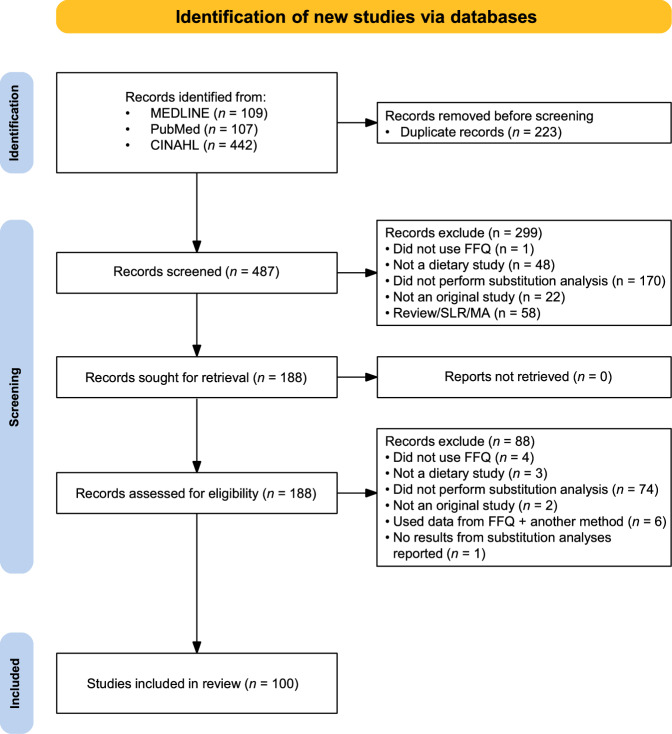
Preferred Reporting Items for Systematic Reviews and Meta-Analyses (PRISMA) flow diagram summarising the process of screening and identification of the literature.

One senior researcher (JL) screened the titles, abstracts and full texts of potentially eligible studies against the eligibility criteria. Studies were included if they: (1) were published in English between January 2018 and December 2024; (2) used only FFQ data in substitution modelling analyses; (3) were original research articles published in peer-reviewed journals. We excluded conference abstracts, reviews, methodological papers and studies that used FFQs but did not conduct substitution analyses. No geographical restrictions were applied. The selection process was documented using a PRISMA flow diagram (Fig. 1).

### Data extraction and synthesis

Using a standardised form in Microsoft Excel, a senior researcher (JL) extracted the following data from each included study: (1) study characteristics [authors, year, country, Science Citation Index (SCI) journal impact quartile]; (2) FFQ details (instrument description, number of items); (3) variables used in substitution modelling; and (4) validation status of FFQ variables and its reporting in the manuscript. For validation status assessment, we specifically examined the nature of comparison methods used, recognising that true validation against a perfect reference method is rarely possible in dietary assessment. We considered studies to have conducted adequate validation if they compared FFQ measurements against methods with different error structures, such as multiple 24-h recalls or weighed food records [11], while acknowledging that most validation studies compare FFQs against other self-reported dietary assessment instruments rather than objective standards and that correlations between methods with shared measurement error may lead to optimistically inflated validation estimates [11, 18, 19].

Validation status was classified as ‘validated’ if the study reported or cited validation studies for the specific FFQ variables used in substitution analyses, ‘partially validated’ if only some variables were validated, or ‘unvalidated’ if no validation information was provided or if validation studies did not cover the variables used in substitution analyses. We specifically examined whether validation studies assessed the accuracy of quantitative estimates, such as percentage differences between the sample mean or median derived from the FFQ and those from the reference method, as this is particularly important for substitution modelling compared to ranking-based analyses. It is important to distinguish between reporting practices and fundamental data quality issues. Poor reporting may mask adequate validation work, but the widespread absence of validation metrics in published substitution analyses represents a critical barrier to evaluating study quality regardless of the underlying validation status. It should also be noted that merely citing a validation study without reporting the specific validation metrics in the manuscript was not considered sufficient for our classification of ‘reporting validation metrics in-text’, as this practice limits readers’ ability to readily assess measurement accuracy of the specific variables used in substitution analyses. Correlation coefficients (Pearson’s *r* or Spearman’s *ρ*) were assigned validity levels as follows: poor (<0.2), fair (0.2–0.4), good (0.4–0.6) and excellent (>0.6). Median and interquartile range values were reported where appropriate throughout the results section to provide more robust measures of central tendency and variability in our validation metrics. Metrics assessing agreement in classification into quantiles, such as weighted kappa, were not included, as they do not directly reflect the ability of the FFQ to provide accurate individual-level intake estimates necessary for substitution modelling. The primary outcome was the prevalence of studies using either unvalidated FFQ variables or variables with poor validity.

## Results

We identified 100 studies [20–119] published between January 2018 and December 2024 that used FFQ data in substitution modelling analyses (Fig. 1). The characteristics of these studies were summarised in Supplementary Table 1. These studies spanned multiple geographical regions, including Australia (*n* = 1) [75], Brazil (*n* = 1) [39], China (*n* = 5) [31, 38, 96, 109, 112], Costa Rica (*n* = 1) [71], Denmark (*n* = 12) [44, 51, 52, 59–61, 63, 72, 81, 86, 101, 110], Finland (*n* = 2) [21, 74], France (*n* = 2) [73, 98], Germany (*n* = 2) [46, 53], Greece (*n* = 1) [58], Iran (*n* = 13) [20, 23, 37, 40, 41, 48, 49, 54, 76, 78, 89, 116, 117], Japan (*n* = 5) [28, 77, 82, 107, 115], Mexico (*n* = 1) [87], the Netherlands (*n* = 15) [22, 29, 30, 62, 69, 79, 83, 85, 90, 93, 94, 100, 102–104], Norway (*n* = 3) [25, 27, 47], Singapore (*n* = 6) [55, 56, 65, 67, 92, 114], South Korea (*n* = 1) [32], Spain (*n* = 4) [26, 34, 35, 88], Sweden (*n* = 1) [45], Switzerland (*n* = 1) [84], the United Kingdom (*n* = 1) [106] and the United States (*n* = 22) [24, 33, 36, 42, 43, 50, 57, 64, 66, 68, 70, 80, 91, 95, 97, 99, 105, 108, 111, 113, 118, 119]. The majority of these studies were prospective cohort studies (*n* = 82) [20, 22, 25, 26, 28–30, 32, 33, 35–53, 55–67, 69, 70, 72–77, 79–81, 84–86, 88, 91–94, 96–99, 101–108, 110–114, 116–119], followed by cross-sectional studies (*n* = 15) [20, 21, 23, 27, 31, 68, 71, 78, 82, 83, 87, 95, 100, 109, 115] and case-control studies (*n* = 4) [24, 34, 54, 89]. Most studies were published in high-impact journals, with 39.0% appearing in SCI Q1 journals [21, 22, 24, 26, 28, 32, 33, 35, 36, 38, 42, 43, 45, 46, 50, 57, 61, 64, 68, 70, 72, 74, 75, 80, 82, 84, 85, 87, 88, 91, 97, 99, 101, 104, 107, 111, 113, 118, 119] and 55.0% in Q2 journals [20, 23, 25, 27, 29, 30, 34, 39–41, 44, 47–49, 51–56, 58–60, 62, 63, 65, 67, 69, 71, 73, 76–79, 81, 86, 89, 90, 92, 93, 96, 98, 100, 102, 103, 105, 106, 108–110, 112, 114, 116, 117]. From these studies, an additional 62 original validation studies [120–181] were cited.

The types of substitution analyses varied across studies, with 7 studies [26, 35, 69, 73, 83, 89, 108] conducting two types. These commonly included macronutrient substitutions (e.g., replacing carbohydrates with fats; *n* = 37) [21–27, 33–35, 37, 38, 41, 45–49, 53, 54, 56, 67, 69, 73, 75, 77, 78, 82, 83, 85, 87–89, 102, 104, 105, 112], nutrients from specific food group substitutions (e.g., replacing animal proteins with plant-based proteins; *n* = 17) [28, 40, 50, 57, 64–66, 69, 73, 83, 89, 99, 103, 108, 115, 118, 119] and specific food item or food group substitutions (e.g., replacing sugar-sweetened beverages with alternatives; *n* = 53) [20, 26, 29–32, 35, 36, 39, 42–44, 51, 52, 55, 58–63, 68, 70–72, 74, 76, 79–81, 84, 86, 90–98, 100, 101, 106–111, 113, 114, 116, 117].

The reporting and quality of FFQ validation varied substantially and revealed significant methodological concerns. More than half of the studies (*n* = 62) did not report relevant validation metrics in the manuscript, but instead only provided a reference to the original validation study (*n* = 58) [21–24, 26, 27, 29, 32, 35–37, 39–43, 45, 46, 48–51, 53, 54, 58–60, 63, 66, 70–72, 74, 75, 77–79, 81, 84, 86–89, 95–97, 99–101, 105–107, 110, 115–119], provided reference to some but not all FFQs used (*n* = 1) [30], or did not provide any reference at all (*n* = 3) [34, 68, 83]. For those that reported validation metrics in-text (*n* = 38) [20, 25, 28, 31, 33, 38, 44, 47, 52, 55–57, 61, 62, 64, 65, 67, 69, 73, 76, 80, 82, 85, 90–94, 98, 102–104, 108, 109, 111–114], 13 only reported a range [38, 44, 52, 56, 65, 66, 69, 82, 104, 108, 111–113], while two studies either reported qualitative statements [25] or quintile agreements [61] with the reference method only.

Delving into the original validation studies revealed further validity information of the FFQs. Correlation coefficients (Spearman’s *ρ* or Pearson’s *r*) between FFQ measurements and reference methods emerged as the predominant validation metric reported in the original validation studies, with 57 reporting that metric [120–123, 125–163, 165, 167–175, 177–180]. While only one of the included studies reported differences in values obtained from the FFQ and the reference method in text [31], 50 original validation studies [120, 122, 123, 126–140, 142–147, 149–153, 155–157, 159–163, 166, 167, 170–180] reported that data. Food records (weighed or estimated) emerged as the most common reference method used (*n* = 31) [120–123, 126, 129, 130, 133, 134, 136, 140, 141, 144, 145, 148, 149, 153–155, 159, 161, 162, 165, 169, 170, 172, 175, 177–180], followed by multiple (up to 12) 24-h recalls (*n* = 23) [125, 128, 131, 138, 139, 142, 143, 146, 147, 150–152, 157, 159, 166–168, 171, 173, 174, 176, 178, 181]. Two included studies [29, 79] utilised an FFQ that was validated against another FFQ only, while another included study using data from multiple cohorts [30] also used data from this FFQ.

For macronutrients, the correlation coefficients exhibited unusually wide ranges: energy intake (0.12–0.77; median, Q1–Q3: 0.43, 0.30 to 0.50), protein intake (0.14–0.64; median, Q1–Q3: 0.44, 0.29 to 0.56), carbohydrate intake (0.25–0.77; median, Q1–Q3: 0.51, 0.41 to 0.66) and total fat intake (0.27–0.72; median, Q1–Q3: 0.53, 0.44 to 0.62). Food group correlations displayed similar variability, ranging from very poor (*r* < 0.20) to excellent (*r* > 0.60). For studies that reported differences from the reference method, no consistent pattern was observed, with some showing minimal differences between the mean values obtained from FFQ *vs*. the reference method, while others showed substantial differences (range –70.3% to +466.7%; median, Q1–Q3: 3.2%, –7.2% to +14.3%).

Of particular concern, more than half of the studies (*n* = 53) conducted substitution analyses using variables that had not been properly validated against reference methods. Among these, 34% were published in SCI Q1 journals [24, 32, 36, 42, 43, 50, 57, 61, 64, 68, 70, 72, 84, 91, 97, 101, 118, 119], while 58.5% appeared in Q2 journals [25, 29, 30, 34, 39, 40, 44, 51, 52, 55, 58–60, 63, 71, 73, 79, 81, 86, 90, 92, 93, 96, 98, 100, 106, 109, 110, 112, 114, 117]. Many of these studies performed substitution analyses with specific food groups (e.g., types of meat, dairy products, or plant-based foods) without reporting validation results for these specific variables. Furthermore, 15 studies [20, 26, 35, 38, 56, 65, 67, 74, 85, 88, 89, 99, 111, 113, 116] conducted substitution analyses where only some variables had been validated. Even when relevant validation metrics were reported, the correlation coefficients were often in the fair to moderate range (*r* = 0.20–0.60). In some cases, the FFQ values substantially deviated from those of the reference method, indicating considerable measurement error in the dietary variables used for substitution modelling.

## Discussion

Our findings reveal a concerning pattern: the majority of substitution modelling analyses that relied on FFQ data had not adequately addressed fundamental limitations in measurement accuracy. This widespread practice raises critical methodological concerns that extend beyond the well-documented limitations of FFQs in nutritional research [7, 8], such as misclassification of dietary patterns, underestimation of portion sizes and failure to capture day-to-day variability in intake.

The core challenge lies in a fundamental mismatch between FFQ capabilities and substitution modelling requirements that extends beyond their intended use to encompass their inherent error structure. FFQs are characterised by systematic measurement errors that represent the primary contributor to total measurement error, as opposed to classical random measurement error patterns [182, 183]. While this systematic error structure may still allow FFQs to detect associations between dietary factors and health outcomes (where ranking individuals along a consumption spectrum suffices) [184, 185], it fundamentally compromises their ability to provide the accurate quantification of absolute amounts required for substitution modelling. The systematic nature of FFQ measurement error means that effect estimates in substitution analyses may be biased in either direction, with the linear error structure affecting both the replaced and replacement foods in ways that compound rather than cancel out [184, 186]. FFQs were designed primarily as ranking tools, optimised for ordering individuals along a spectrum of dietary intake [15, 187]. Traditional nutritional analyses respect this design by focusing on relative comparisons, such as comparing health outcomes between the lowest and highest quartiles of consumption. However, substitution modelling demands something fundamentally different: accurate quantification of absolute amounts of foods being replaced [12].

This creates two distinct methodological problems. First, substitution models require precise measurement of both the replaced and replacement foods. Using data from FFQ, which is known to be unable to produce precise individual-level estimates, introduces compounded measurement error that exceeds what is typically encountered in analyses of single dietary components. Second, the assumption of linear substitution effects becomes particularly questionable when working with FFQ data, given the known difficulties in accurate portion size estimation and the semi-quantitative nature of most FFQs [7] and the consistency assumption required for causal inference [184, 186, 188].

It is important to recognise that not all limitations stem from poor questionnaire design or choice of reference method. Even well-developed FFQs evaluated against high-quality biomarkers demonstrate systematic biases, particularly correlated errors linked to true intake [189, 190]. This error structure means that FFQs can distort associations in substitution models, not merely attenuate them, raising concerns about whether the method is fit for such purposes rather than only reflecting flaws in development.

Despite these fundamental challenges, researchers often proceed with substitution analyses under the incorrect assumption that FFQs provide accurate estimates of individual dietary intake, as shown in our review. This assumption persists despite extensive evidence that FFQs are primarily designed for ranking individuals [7, 8] rather than quantifying precise intakes [15, 187]. The consequences of this misalignment between tool capabilities and analytical requirements warrant careful consideration, particularly as findings from these studies increasingly inform dietary guidelines and public health recommendations [12].

The validity of these estimated substitution effects becomes questionable when components are inaccurately measured, as demonstrated by research using recovery biomarkers such as doubly labelled water for energy intake and urinary nitrogen for protein intake. These objective measures reveal that FFQs consistently misreport ‘true’ dietary intake with systematic rather than random error patterns [191–195]. For instance, Schatzkin et al.[191] found that energy intake reported through FFQs can be approximately 30% lower compared to measurements using doubly labelled water. However, the magnitude of underestimation varies by nutrient, with studies showing that compared to energy intake, the underestimation was considerably less for potassium and protein, especially when modelled as a ratio over energy [187, 196]. Similar patterns of differential systematic underestimation have been documented for dietary fat intakes, though the variability in error patterns across nutrients creates additional challenges for substitution modelling where multiple dietary components must be accurately quantified simultaneously. Our analysis of validation metrics across studies revealed wide-ranging correlation coefficients, with many studies reporting only fair to moderate correlations (*r* = 0.2–0.6) between FFQ measurements and reference methods. The deviations from reference values were highly variable, with some nutrients/food groups underestimated by 70% and others overestimated by over 450%. We acknowledge that the modest correlations between FFQs and reference methods reflect limitations of both assessment approaches, as most validation studies compare FFQs against other self-reported dietary assessment instruments, such as 24-h recalls and food records. Any relationship between two measurements prone to self-reported bias leads to optimistically inflated validation coefficients, since errors tend to be correlated between self-reported methods [11, 18, 19]. This means that the reported correlation coefficients likely overestimate true validity, making the validation problem in substitution modelling potentially more severe than our findings suggest. Reference methods like 24-h recalls and food records, while providing more detailed intake data, have their own limitations in capturing long-term dietary habits. This mutual imperfection in dietary assessment tools highlights the fundamental challenge of measuring true dietary intake, particularly for substitution modelling, which requires accurate absolute quantification rather than merely correct ranking.

This measurement error is particularly problematic as it is not merely random but often systematic, potentially leading to biased estimates in substitution analyses [197]. Random errors, if left unaddressed, can also produce bias [183], though they can be mitigated with statistical methods, unlike systematic errors, which are much harder to correct. For FFQ data, systematic errors are typically the dominant source of total error and the error structure departs from classical measurement error assumptions, making standard correction methods insufficient for substitution modelling [182, 183]. The challenge is further compounded by the precision limitations of quantity estimates in FFQs, as most studies employ semi-quantitative FFQs that rely on standardised serving sizes derived from national survey data [11]. While these standardised portions are intended to be adjusted to reflect actual consumption, the consistency and accuracy of such adjustments are questionable [7, 11], with studies comparing FFQs to food records demonstrating that quantifying intake introduces substantial additional error beyond merely capturing food choices [187, 193, 194]. Memory limitations also significantly contribute to inaccuracies when recalling dietary patterns over extended periods [198, 199], leading to substantial recollection biases [200, 201] that further compound the systematic error patterns characteristic of FFQs. Substitution modelling is especially vulnerable to these issues, as it directly estimates the effect of replacing one dietary component with another, placing greater demands on measurement accuracy than general compositional analyses [9, 10, 12].

The widespread use of unvalidated variables in substitution analyses represents another critical methodological concern. Our review found that more than half of the studies conducted substitution modelling without properly validating their dietary variables against reference methods, despite clear evidence that FFQ measurement accuracy varies substantially across food groups. This validation gap is particularly concerning given documented evidence that certain commonly substituted food groups show poor measurement validity. Research by Traynor et al. [202] demonstrated this problem clearly: when comparing FFQ data with 24-h recalls, they found only fair to moderate agreement for total fruit and vegetable intake (*ρ* = 0.41) and even poorer agreement for individual vegetable types (*ρ* < 0.4). Similar validity issues have been consistently reported for other frequently substituted foods, with vegetables, added fats and sweets showing particularly poor measurement accuracy [203–205]. These findings suggest that many substitution analyses may be based on fundamentally unreliable measurements of the very dietary components they aim to study.

More accurate dietary assessment methods include multiple-day food records and 24-h recalls, which can be used to estimate long-term dietary habits when replicate measures are available and appropriate measurement error correction methods such as regression calibration and multiple imputation are applied [206]. However, these approaches face practical constraints of higher participant burden and increased costs compared to FFQs [11]. Emerging technologies offer promising middle-ground solutions - mobile phone-based tools and image-based dietary assessment methods may combine FFQs’ practicality with enhanced measurement precision [207, 208]. For researchers who must rely on FFQ data, several methodological approaches, including the application of appropriate statistical techniques, can strengthen the validity of their analyses. Calibration studies using recovery biomarkers can adjust for systematic measurement error, though this approach is currently limited to nutrients with available recovery biomarkers such as energy, protein, potassium and sodium [192, 209, 210]. To address measurement error, researchers can employ statistical techniques like regression calibration and multiple imputation [188, 211], though these methods typically address random measurement error patterns in data from short-term instruments such as 24-h recalls and food records when estimating usual intakes, where random errors represent a larger proportion of total measurement error [188]. The systematic errors characteristic of FFQs, however, cannot be easily eliminated through statistical approaches alone. Additionally, structural equation modelling provides tools for accounting for correlated errors across dietary components and the mediation effect of lurking variables [212].

Looking ahead, three key priorities emerge from our findings. Most critically, the field needs specialised dietary assessment tools designed specifically for substitution analyses - tools that optimise the accuracy of the results of quantitative analysis while remaining practically feasible. Second, appropriate statistical methods must be used to address the unique patterns of measurement error in substitution modelling, including Bayesian methods, particularly when working with FFQ data. Additionally, while different analytical frameworks may better account for and acknowledge measurement uncertainty in substitution modelling, it is important to recognise that no statistical approach can recover from or eliminate systematic measurement error in the underlying FFQ data [188, 213]. Once dietary data are measured with systematic error, statistical methods cannot restore the missing accuracy unless additional data sources, such as recovery biomarkers, are incorporated to calibrate the measurements [214]. Finally, the establishment of standardised reporting guidelines for substitution analyses would ensure consistent documentation of validation methods and results across studies. Notably, we observed that FFQs in the reviewed studies were frequently labelled as ‘validated’ without clarification of the variables for which they were validated or the validation metrics used. The lack of specificity is misleading, potentially weakening the findings and causing confusion about whether the FFQ is suitable for the analysis. Editors and reviewers must recognise that describing an FFQ as ‘validated’ does not automatically imply its suitability for the variables under investigation. A more transparent and precise approach to reporting FFQ validation is imperative to ensure clarity and scientific rigor.

Our review offers several methodological advances in understanding the challenges of substitution modelling. First, by examining FFQ validation practices across 100 studies spanning multiple geographical regions and research contexts, we provide the first comprehensive assessment of inappropriate use of unvalidated FFQ data in substitution analyses worldwide. This global perspective reveals consistent methodological concerns that transcend regional differences in dietary assessment practices. Second, our detailed analysis revealed heterogeneity in measurement accuracy, which has particular implications for substitution modelling, where errors in measuring both the replaced and replacement foods can compound to significantly bias effect estimates. Thus, our review provided crucial evidence for improving the methodological rigor of future substitution analyses.

A particular limitation of our review is that we could not quantify the relative impact of measurement error under different scenarios and settings. While we argue that measurement error is particularly problematic in substitution modelling, future research should systematically evaluate how different magnitudes and patterns of measurement error affect model conclusions through simulation studies and direct comparisons of substitution analyses using different dietary assessment methods within the same population. Furthermore, our review focused primarily on the validation aspects of FFQ data in substitution modelling and we did not extensively examine the various statistical approaches used in the substitution analyses themselves. Furthermore, our review focused primarily on the validation aspects of FFQ data in substitution modelling and we did not extensively examine the various statistical approaches used in the substitution analyses themselves. Additionally, while we focused our discussion on the correlation between FFQs and reference methods, it is important to note that correlation and agreement are not equivalent [215].

## Conclusions

Our findings demonstrate that while FFQ-based substitution modelling is widely used in nutritional epidemiology, significant methodological limitations raise concerns about the validity of conclusions drawn from these studies. The combination of substantial measurement error, use of unvalidated variables and the particular challenges of quantifying both the replaced and replacement foods suggests that findings from such analyses should be interpreted with considerable caution. Until improved dietary assessment methods and/or appropriate statistical methods are used for substitution analyses, researchers should carefully consider whether FFQ data are appropriate for their intended substitution modelling approach.

## Supplementary information

Supplementary Table 1
