# Supplementary material for: The (ab)use of food frequency questionnaire data in substitution modelling in nutritional epidemiology: a critique
Source: Eur J Clin Nutr. 2026 Feb 22;80(5):458–68. doi: 10.1038/s41430-026-01712-7 (PMC13186700; doi:10.1038/s41430-026-01712-7)
Supplement: Supplementary file 1 — Supplementary Table 1 [file 41430_2026_1712_MOESM1_ESM.pdf]

						Validity data											
									^Unadjusted r or ρ			^Adjusted r or ρ			Difference from reference method		
Ref, Country	Study design	SCI-Q	FFQ details	Validation metrics reported in manuscript?	†Substitution examined	Ref	*n* in validation study and reference method	Variable	Male	Female	Total	Male	Female	Total	Male	Female	Total
Australia																	
Melaku *et al*. 2019	Prospective cohort	Q1	DQES-V3.1 121-item, semi-quantitative	N	Macronutrients with each other	Hodge *et al.* 2000	63 females; 7d WFR	Energy	-	0.23	-	-	-	-	-	6.0%	-
								Protein	-	0.36	-	-	0.32	-	-	1.4%	-
								CHO	-	0.37	-	-	0.7	-	-	17.6%	-
								Sugars	-	0.45	-	-	0.66	-	-	21.2%	-
								Starch	-	0.35	-	-	0.29	-	-	10.6%	-
								Fat	-	0.36	-	-	0.68	-	-	-2.4%	-
								SFA	-	0.36	-	-	0.59	-	-	5.4%	-
								PUFA	-	0.52	-	-	0.55	-	-	-9.7%	-
								MUFA	-	0.41	-	-	0.59	-	-	-8.1%	-
								Alcohol	-	0.58	-	-	0.61	-	-	-23.9%	-
								Fibre	-	0.47	-	-	0.66	-	-	23.4%	-
Brazil																	
Ferreira *et al.* 2024	Prospective cohort	Q2	114-item semi-quantitative	N	Unprocessed/Minimally processed food (NOVA-1) and culinary ingredients (NOVA-2) for ultra-processed foods (NOVA-4)	Molina *et al.* 2013	136 males + 145 females; 2 x 3d FR	Energy	-	-	-	-	-	-	-	-	36.2%
								Protein	-	-	-	-	-	-	-	-	38.8%
								CHO	-	-	-	-	-	-	-	-	43.6%
								Fat	-	-	-	-	-	-	-	-	26.4%
								Fibre	-	-	-	-	-	-	-	-	78.3%
China																	
Chiu *et al.* 2018	Cross-sectional	-	64-item quantitative	Y, specific ρ	Meat or fish for soy<br>Refined grains, fruit or fruit juice for whole grains	Reported in-text	No information provided for no. of participants	Energy	-	-	0.58	-	-	-	-	-	3.4%
								Protein	-	-	0.50	-	-	0.49	-	-	-5.8%
						Chiu *et al.* 2014	21 males and 57 females; 3d FR	CHO	-	-	0.66	-	-	0.44	-	-	7.6%*
								Fat	-	-	0.30	-	-	0.44	-	-	-8.9%
								SFA	-	-	0.42	-	-	0.46	-	-	-5.0%
								PUFA	-	-	0.17	-	-	0.15	-	-	-17.2%*
								MUFA	-	-	0.41	-	-	0.44	-	-	-15.7%*
								Fibre	-	-	0.47	-	-	0.55	-	-	-19.4%
								Soy	-	-	0.41	-	-	-	-	-	-
								Meat	-	-	0.46	-	-	-	-	-	-
								Fish	-	-	0.55	-	-	-	-	-	-
								Eggs	-	-	0.47	-	-	-	-	-	-
								Dairy	-	-	0.39	-	-	-	-	-	-
								Vegetables	-	-	0.47	-	-	-	-	-	-
								Fruits	-	-	0.30	-	-	-	-	-	-
								Grains	-	-	0.68	-	-	-	-	-	-
Fan *et al.* 2024	Prospective cohort	Q1	120-item semi-quantitative	Y, range of ρ	Different types of FAs for SFA	None cited; reported in-text only	No information provided for no. of participants; FR and erythrocyte FA	Macronutrients	-	-	0.56-0.74	-	-	-	-	-	-
								SFA	-	-	0.29	-	-	-	-	-	-
								PUFA	-	-	0.37	-	-	-	-	-	-
Sun *et al.* 2023	Prospective cohort	Q2	266-item quantitative	N	Wholegrain for refined grain	Woo *et al.* 1997	500 males + 510 females; 1 x 24HR	Energy	-	-	-	-	-	-	2.2%	-4.6%	-
Yang *et al.* 2022a	Prospective cohort	Q2	107-item semi-quantitative	Y, specific r for protein and range for other nutrients	Protein for fat or CHO	Cheng *et al.* 2008	125 females; 6 x 24HR	Energy	-	‡0.68	-	-	-	-	-	11.9%	-
								Protein	-	‡0.61	-	-	‡0.22	-	-	8.2%	-
								CHO	-	‡0.68	-	-	‡0.49	-	-	10.5%	-
								Fat	-	‡0.55	-	-	‡0.49	-	-	24.9%	-
								Fibre	-	‡0.63	-	-	‡0.34	-	-	10.2%	-
Wu *et al.* 2022	Cross-sectional	Q2	Inadequate information provided	Y, specific ρ for energy only	Fresh vegetables for preserved vegetables	Reported in text only	200 males + females; 2 x 24HR	Energy	-	-	0.60	-	-	-	-	-	-
Costa Rica																	
Luan *et al.* 2020	Cross-sectional	Q2	135-item semi-quantitative	N	Alternative protein (fish, chicken without skin or fat, chicken with skin and fat, low fat milk, total milk and legumes) for red meat	Kabagambe *et al.* 2001	78 males + 42 females; 7 x 24HR	Energy	-	-	-	-	-		-	-	25.5%
								Protein	-	-	‡0.39	-	-	‡0.46	-	-	16.9%
								CHO	-	-	‡0.47	-	-	‡0.50	-	-	17.1%
								Fat	-	-	‡0.59	-	-	‡0.74	-	-	50.8%
								SFA	-	-	‡0.58	-	-	‡0.71	-	-	55.0%
								MUFA	-	-	‡0.49	-	-	‡0.64	-	-	54.5%
								PUFA	-	-	‡0.54	-	-	‡0.75	-	-	50.0%
								Fibre	-	-	‡0.65	-	-	‡0.72	-	-	25.0%
Denmark																	
Nielsen *et al.* 2022	Prospective cohort	Q2	192-item semi-quantitative	N	Fish, poultry and red meat with each other	Tjønneland *et al.* 1991	59 males + 85 females; 2 x 7d WFR	Energy	0.40	0.23	-	-	-	-	-1.8%	-2.1%	-
Ibsen *et al.* 2019	Prospective cohort	Q2		Y, range of ρ				Protein	0.41	0.14	-	0.52	0.26	-	-7.4%	-8.2%	-
Lasota *et al.* 2019	Prospective cohort	Q2		N				CHO	0.44	0.34	-	0.40	0.47	-	13.9%	22.8%	-
Venø *et al.* 2018	Prospective cohort	Q1		N				Fat	0.54	0.27	-	0.67	0.48	-	-13.2%	-13.2%	-
Hansen *et al.* 2021	Prospective cohort	Q2		Y, range of ρ	Potatoes with other vegetables; Substitutions between subgroups of other vegetables			SFA	0.42	0.26	-	0.46	0.39	-	-3.6%	-6.3%	-
Wurtz *et al.* 2021a	Prospective cohort	Q2		N				PUFA	0.53	0.28	-	0.60	0.31	-	-7.2%	3.1%	-
Pokharel *et al.* 2024	Prospective cohort	Q2		N													
Laursen *et al.* 2018	Prospective cohort	Q1		Y, quintile agreement	Dairy products with each other												
Ibsen *et al.* 2021	Prospective cohort	Q2		N													
Kvist *et al.* 2020	Prospective cohort	Q2		N													
Laursen *et al.* 2022	Prospective cohort	Q2		N													
Lyskjaer *et al.* 2020	Prospective cohort	Q1		N	Oatneal, eggs, yoghurt and white bread for each other												
Finland																	
Ahola *et al.* 2018	Cross-sectional	Q1	19-item qualitative	N	Macronutrients with each other	Ahola *et al.* 2017	238 males + 304 females; 2 x 3d FR	Energy	-	-	0.73	-	-	-	-	-	-
								Other macronutrients	-	-	>0.43	-	-	-	-	-	-
Maukonen *et al.* 2023	Prospective cohort	Q1	110-item semi-quantitative	N	Red meat or processed meat with plant-based foods (legumes, vegetables, fruits, cereals, or a combination of these)	Männistö *et al.* 1996	152 females; 2 x 7d FR	Energy	-	-	‡0.23	-	-	-	-	-	-
								Protein	-	-	‡0.23	-	-	‡0.63	-	-	-
								CHO	-	-	‡0.25	-	-	‡0.49	-	-	-
								Fat	-	-	‡0.30	-	-	‡0.44	-	-	-
								SFA	-	-	‡0.37	-	-	‡0.59	-	-	-
								Beef, pork	-	-	‡0.37	-	-	‡0.42	-	-	-
								Poultry	-	-	‡0.45	-	-	‡0.54	-	-	-
								Sausages	-	-	‡0.47	-	-	‡0.61	-	-	-
								Vegetables	-	-	‡0.60	-	-	‡0.70	-	-	-
								Fruits	-	-	‡0.62	-	-	‡0.70	-	-	-
								Wheat products	-	-	‡0.45	-	-	‡0.62	-	-	-
								Rye products	-	-	‡0.37	-	-	‡0.48	-	-	-
France																	
MacDonald *et al.* 2023	Prospective cohort	Q2	208-item semi-quantitative	Y, specific ρ	MUFA, PUFA, CHO, and animal or vegetable protein for SFA	van Liere *et al.* 1997	119 females; 9-12 x 24HR	Energy	-	-	0.40	-	-	-	-	38.1%*	-
Thao *et al.* 2023	Prospective cohort	Q2		Y, specific ρ	Processed red meat, non-processed red meat, poultry, fish, eggs and vegetables for each other			Protein	-	-	0.29	-	-	0.56	-	40.8%*	-
								CHO	-	-	0.42	-	-	0.64	-	27.6%*	-
								Fat	-	-	0.49	-	-	0.49	-	47.3%*	-
Germany																	
Inan-Eroglu *et al.* 2024	Prospective cohort	Q2	105 to 149-item semi-quantitative	N	Total protein for CHO	Kroke *et al.* 1999	75 males + 59 females; 3 x 24HR + urinary nitrogen (protein) + DLW (energy)	Energy	-	-	‡0.48	-	-	-	-	-	-22.2%
								Protein	-	-	‡0.41	-	-	-	-	-	-22.7%
								CHO	-	-	‡0.56	-	-	‡0.54	-	-	8.6%
								Fat	-	-	‡0.59	-	-	‡0.71	-	-	-4.2%
								SFA	-	-	‡0.62	-	-	‡0.70	-	-	-10.9%
								MUFA	-	-	‡0.59	-	-	‡0.71	-	-	-15.5%
								PUFA	-	-	‡0.47	-	-	‡0.60	-	-	1.7%
								Fibre	-	-	‡0.47	-	-	‡0.50	-	-	24.3%
Harris *et al.* 2018	Prospective cohort	Q1	80-item quantitative	N	CHO with different FAs	None cited in text; Steigler *et al.* 2010	52 males + 49 females; 1 x 24HR	Energy	-	-	-	-	-	-	-	-	9.6%
								Protein	-	-	-	-	-	-	-	-	8.3%
								CHO	-	-	-	-	-	-	-	-	20.6%
								Fat	-	-	-	-	-	-	-	-	1.6%
								SFA	-	-	-	-	-	-	-	-	8.0%
								MUFA	-	-	-	-	-	-	-	-	0.0%
								LA	-	-	-	-	-	-	-	-	14.3%
								AA	-	-	-	-	-	-	-	-	83.1%
								EPA	-	-	-	-	-	-	-	-	466.7%
								DHA	-	-	-	-	-	-	-	-	151.7%
Greece																	
Kouvari *et al.* 2024	Prospective cohort	Q2	156-item semi-quantitative	N	Dairy product sub-types for each other	Katsouyyanni *et al.* 1997	42 males + 38 females; 12 x 24HR + biomarkers	Energy	0.63	0.46	-	0.66	0.50	-	7.3%	31.6%	-
								Protein	0.67	0.54	-	0.56	0.44	-	-7.4%	0.6%	-
								SFA	0.68	0.49	-	0.47	0.49	-	4.6%	15.5%	-
								MUFA	0.64	0.41	-	0.29	0.41	-	26.4%	68.4%	-
								PUFA	0.56	0.24	-	0.15	0.21	-	56.0%	58.6%	-
								Fibre	0.27	0.31	-	0.41	0.19	-	27.8%	93.0%	-
Iran																	
Mirmiran *et al.* 2020	Prospective cohort	Q2	168-item semi-quantitative	Y, specific r	Total red and processed meat with low-fat dairy, nuts, wholegrains and legumes	Mirmiran *et al.* [illegible]	61 males + 71 females; 12 x [illegible]	Energy	‡0.55	‡0.46	-	‡0.55	‡0.46	-	12.0%	26.0%	-

Ref, Country	Study design	SCI-Q	FFQ details	Validation metrics reported in manuscript?	[†]Substitution examined	Validity data			^Unadjusted r or ρ			^Adjusted r or ρ			Difference from reference method		
						Ref	*n* in validation study and reference method	Variable	Male	Female	Total	Male	Female	Total	Male	Female	Total
Aghayan *et al.* 2019	Cross-sectional	Q2		Y, specific r for nuts only	Nuts for sweet, salty, and energy-dense nutrient-poor solid snacks	2010	24HR	Protein	[‡]0.64	[‡]0.48	-	[‡]0.65	[‡]0.50	-	5.0%	21.0%	-
Amirkalali *et al.* 2021	Cross-sectional	Q2		N	Macronutrients with each other			CHO	[‡]0.38	[‡]0.47	-	[‡]0.39	[‡]0.47	-	8.0%	21.0%	-
Hosseini-Esfahani *et al.* 2019	Prospective cohort	Q2		N				Fat	[‡]0.62	[‡]0.40	-	[‡]0.59	[‡]0.38	-	11.0%	30.0%	-
Gaeini *et al.* 2021	Prospective cohort	Q2		N				SFA	[‡]0.61	[‡]0.37	-	[‡]0.58	[‡]0.34	-	11.0%	32.0%	-
Moslehi *et al.* 2024	Cross-sectional	Q2		N				PUFA	[‡]0.37	[‡]0.35	-	[‡]0.33	[‡]0.32	-	7.0%	26.0%	-
Esfandiar *et al.* 2021	Prospective cohort	Q3		N				MUFA	[‡]0.55	[‡]0.39	-	[‡]0.49	[‡]0.34	-	6.0%	28.0%	-
Hosseini-Esfahani *et al.* 2020	Prospective cohort	Q2		N				Fibre	[‡]0.68	[‡]0.61	-	[‡]0.67	[‡]0.60	-	9.0%	-17.0%	-
Jahromi *et al.* 2024	Case-control	Q2		N		Esfahani *et al.* 2010		Meat	0.46	0.45	-	0.37	0.36	-	-15.0%	-3.0%	-
Sasanfar *et al.* 2022	Case-control	Q2		N	Macronutrients with each other; plant fat or protein with animal counterparts			Dairy	0.89	0.61	-	0.53	0.61	-	39.0%	35.0%	-
Yuzbashian *et al.* 2021a	Prospective cohort	Q2		N	Nuts, legumes, wholegrains, poultry and fish, egg, or red and processed meat for dairy			Nuts and seeds	0.53	0.28	-	0.58	0.27	-	35.0%	74.0%	-
Yuzbashian *et al.* 2021b	Prospective cohort	Q2		N	Dairy product subgroups for each other			Wholegrains	0.45	0.49	-	0.45	0.46	-	7.0%	17.0%	-
								Legumes	0.38	0.09	-	0.43	0.10	-	-23.0%	-27.0%	-
								Simple sugars	0.91	0.74	-	0.87	0.65	-	-1.0%	-11.0%	-
								Salty snacks	0.18	0.30	-	0.23	0.35	-	2.0%	35.0%	-
Forootani *et al.* 2024	Prospective cohort	Q2	178-item semi-quantitative	N	Plant protein for animal protein	Zimorovat *et al.* 2022	89 males + 91 females; 9 x 3d WFR	Energy	-	-	[‡]0.41	-	-	[‡]0.26	-	-	35.4%
								Protein	-	-	[‡]0.42	-	-	[‡]0.29	-	-	74.5%
								CHO	-	-	[‡]0.41	-	-	[‡]0.28	-	-	30.6%
								Fat	-	-	[‡]0.38	-	-	[‡]0.34	-	-	36.6%
Japan																	
Budhathoki *et al.* 2019	Prospective cohort	Q1	138-item semiquantitative	Y, specific ρ	Plant protein for red meat protein; Plant protein for processed meat protein	Nanri *et al.* 2015	102 males + 113 females; 28d FR	Animal protein	0.21	0.26	-	-	-	-	-	-	-
								Plant protein	0.59	0.49	-	-	-	-	-	-	-
Yoshioka *et al.* 2023	Cross-sectional	Q3	FFQg version 5; 47-item semi-quantitative	N	Animal protein with plant protein	Takahashi *et al.* 2001	66 males and females; 7d FR	Energy	-	-	0.47	-	-	-	-	-	6.8%
								Protein	-	-	0.42	-	-	-	-	-	3.3%
								Meat/processed meat	-	-	0.57	-	-	-	-	-	30.4%*
								Fish/seafood	-	-	0.27	-	-	-	-	-	-6.9%
								Eggs	-	-	0.48	-	-	-	-	-	-4.9%
								Milk	-	-	0.72	-	-	-	-	-	13.0%
								Other dairy products	-	-	0.58	-	-	-	-	-	-16.5%
								Small fish	-	-	0.22	-	-	-	-	-	-14.3%
								Bread	-	-	0.76	-	-	-	-	-	-14.2%*
								Rice	-	-	0.66	-	-	-	-	-	11.4%*
								Noodles	-	-	0.48	-	-	-	-	-	-11.6%
								Soybean/soy products (excluding miso)	-	-	0.53	-	-	-	-	-	39.7%*
								Seaweed	-	-	0.30	-	-	-	-	-	-58.1%*
								Potatoes	-	-	0.32	-	-	-	-	-	-8.3%
								Nuts and seeds	-	-	0.23	-	-	-	-	-	-47.1%
								Miso	-	-	0.68	-	-	-	-	-	6.8%
Okuda and Sasaki, 2024	Cross-sectional	Q1	BDHQ15y 90-item semi-quantitative	Y, range of r	Other amino acids with leucine	**Adults:** Suga *et al.* 2018 **Adolescents**: Okuda *et al.* 2019	**Adults:** 92 males + 92 females; 4d semi-WFR **Adolescents:** 118 males + 130 females; urinary creatinine	Energy	0.41	0.30	-	-	-	-	-	-	-
								Protein (adolescents)	-	-	0.12 to 0.25	-	-	0.11 to 0.30	-	-	-
								Amino acids (adults)	0.29 to 0.40	0.24 to 0.32	-	0.21 to 0.38	0.35 to 0.58	-	-	-	-
Morisaki *et al.* 2018	Prospective cohort	Q2	172-item semi-quantitative	N	Protein with fat or CHO	Yokoyama *et al.* 2016	98 males + 142 females; 12d WFR	Energy	-	-	-	0.45	0.17	-	3.2%	12.8%	-
								Protein	-	-	-	0.40	0.33	-	-5.9%	9.6%	-
								CHO	-	-	-	0.74	0.40	-	3.2%	11.4%	-
								Fat	-	-	-	0.53	0.33	-	-2.4%	18.1%	-
								SFA	-	-	-	0.48	0.46	-	2.3%	25.0%	-
								MUFA	-	-	-	0.55	0.21	-	-0.9%	22.9%	-
								PUFA	-	-	-	0.50	0.28	-	0.7%	20.5%	-
								Fibre	-	-	-	0.66	0.61	-	-13.7%	6.7%	-
Watanabe *et al.* 2024	Prospective cohort	Q1	40-item semi-quantitative	N	Processed meat, chicken, fish, eggs, dairy products, and pulses for red meat	Date *et al.* 2005	85 males + females; 4 x 3d WFR	Energy	-	-	0.20	-	-	-	-	-	-
								Protein	-	-	0.24	-	-	0.24	-	-	-38.6%
								Fat	-	-	0.28	-	-	0.46	-	-	-44.4%
								Ham or sausages	-	-	0.67	-	-	-	-	-	-
								Chicken	-	-	0.53	-	-	-	-	-	-
								Fresh fish	-	-	0.54	-	-	-	-	-	-
								Dried fish or salted fish	-	-	0.46	-	-	-	-	-	-
								Milk	-	-	0.69	-	-	-	-	-	-
								Cheese	-	-	0.57	-	-	-	-	-	-
								Yoghurt	-	-	0.54	-	-	-	-	-	-
								Butter	-	-	0.55	-	-	-	-	-	-
								Beef	-	-	0.59	-	-	-	-	-	-
								Pork	-	-	0.44	-	-	-	-	-	-
								Boiled beans	-	-	0.59	-	-	-	-	-	-
Mexico																	
Rivera-Paradez *et al.* 2023	Cross-sectional	Q1	85-item semi-quantitative	N	Macronutrients for each other	Hernández-Avila *et al.* 1998	134 females; 16 x 24HR	Energy	-	[‡]0.50	-	-	-	-	-	22.1%	-
								Protein	-	[‡]0.42	-	-	[‡]0.21	-	-	25.7%	-
								CHO	-	[‡]0.51	-	-	[‡]0.49	-	-	33.6%	-
								Fat	-	[‡]0.50	-	-	[‡]0.45	-	-	-4.5%	-
								SFA	-	[‡]0.46	-	-	[‡]0.51	-	-	14.3%	-
								MUFA	-	[‡]0.49	-	-	[‡]0.46	-	-	0.0%	-
								PUFA	-	[‡]0.42	-	-	[‡]0.07	-	-	-53.1%	-
								Fibre	-	[‡]0.47	-	-	[‡]0.46	-	-	42.2%	-
Netherlands																	
Buso *et al.* 2022	Prospective cohort	Q2	Flower FFQ; 110-item semi-quantitative	N	LNCB for SSB; Fruit juice for SSB; water for SSB or LNCB	Brouwer-Brolsma *et al.* 2022	191 males + 210 females; a validated regular FFQ	SSB	-	-	0.58	-	-	-	-	-	35.0%
Naomi *et al.* 2023	Prospective cohort	Q2		N				LNCB	-	-	0.47	-	-	-	-	-	17.4%
								Water	-	-	0.07	-	-	-	-	-	2153.0%
								Fruit juice	-	-	0.65	-	-	-	-	-	-6.0%
Van Eekelen *et al.* 2019	Cross-sectional	Q2	104-item semi--quantitative	N	Alcoholic beverages with nonalcoholic beverages	Verkleij-Hagoort *et al.* 2007	53 females; 3 x 24HR	Energy	-	[‡]0.45	-	-	-	-	-	5.8%	-
								Protein	-	[‡]0.55	-	-	[‡]0.40	-	-	-4.3%	-
								CHO	-	[‡]0.31	-	-	[‡]0.36	-	-	6.1%	-
								Fat	-	[‡]0.53	-	-	[‡]0.35	-	-	18.2%	-
Voortman *et al.* 2021	Prospective cohort	Q1	170-item semi-quantitative	Y, range of r	Macronutrients with each other	Feunekes *et al.* 1993	95 males + 96 females; DHI + erythrocyte membrane FA (biomarker)	Energy	[‡]0.77	[‡]0.76	[‡]0.83	-	-	-	-	-	3.4%
Verspoor *et al.* 2020	Prospective cohort	Q2		Y, specific r	Fat or protein for CHO; protein for fat			Fat	[‡]0.71	[‡]0.74	[‡]0.78	-	-	-	-	-	10.8%
								SFA	[‡]0.68	[‡]0.74	[‡]0.75	-	-	-	-	-	9.3%
								MUFA	[‡]0.71	[‡]0.75	[‡]0.78	-	-	-	-	-	8.6%
								PUFA	[‡]0.53	[‡]0.58	[‡]0.61	-	-	-	-	-	19.6%
								LA	[‡]0.48	[‡]0.53	[‡]0.56	-	-	-	-	-	22.4%
								PUFA (biomarker)	-	-	[‡]0.33	-	-	-	-	-	76.9%
								LA (biomarker)	-	-	[‡]0.44	-	-	-	-	-	-41.6%
						Klipstein-Grobusch *et al.* 1998	39 males + 41 females; 15d FR	Energy	-	-	[‡]0.69	-	-	-	-	-	4.1%
								Protein	-	-	[‡]0.73	-	-	[‡]0.63	-	-	13.1%
								CHO	-	-	[‡]0.72	-	-	[‡]0.77	-	-	3.0%
								Fat	-	-	[‡]0.57	-	-	[‡]0.48	-	-	1.6%
								SFA	-	-	[‡]0.49	-	-	[‡]0.39	-	-	1.2%
								MUFA	-	-	[‡]0.56	-	-	[‡]0.49	-	-	-1.7%
								PUFA	-	-	[‡]0.57	-	-	[‡]0.52	-	-	11.0%
								LA	-	-	[‡]0.54	-	-	[‡]0.52	-	-	27.6%
								Fibre	-	-	[‡]0.64	-	-	[‡]0.59	-	-	41.5%
			389 item semi-quantitative			Goldbohm *et al.* 1994	59 males + 50 females; 9d FR	Energy	-	-	[‡]0.74	-	-	-	-	-	-14.5%
								Protein	-	-	[‡]0.61	-	-	[‡]0.59	-	-	-11.5%
								CHO	-	-	[‡]0.77	-	-	[‡]0.71	-	-	-11.7%
								Fat	-	-	[‡]0.72	-	-	[‡]0.52	-	-	-17.7%
								SFA	-	-	[‡]0.73	-	-	[‡]0.58	-	-	-23.2%
								PUFA	-	-	[‡]0.73	-	-	[‡]0.75	-	-	4.7%
								Fibre	-	-	[‡]0.74	-	-	[‡]0.74	-	-	6.2%
Oosterwijk *et al.* 2019	Cross-sectional	-	177-item semiquantitative	N	Macronutrients for each other; Animal protein with plant protein	None cited	No information provided	None provided	-	-	-	-	-	-	-	-	-
Pertiwi *et al.* 2020	Prospective cohort	Q1	204-item semi-quantitative	Y, specific r for LA	dietary LA for the sum of saturated (SFA) and trans fatty acids (TFA)	Feunekes *et al*. 1993	95 males + 96 females; DHI + erythrocyte membrane FA (biomarker)	Energy	[‡]0.77	[‡]0.76	[‡]0.83	-	-	-	-	-	3.4%
								Fat	[‡]0.71	[‡]0.74	[‡]0.78	-	-	-	-	-	10.8%
								SFA	[‡]0.68	[‡]0.74	[‡]0.75	-	-	-	-	-	9.3%
								MUFA	[‡]0.71	[‡]0.75	[‡]0.78	-	-	-	-	-	8.6%
								PUFA	[‡]0.53	[‡]0.58	[‡]0.61	-	-	-	-	-	19.6%
								LA	[‡]0.48	[‡]0.53	[‡]0.56	-	-	-	-	-	22.4%
								PUFA (biomarker)	-	-	[‡]0.33	-	-	-	-	-	76.9
								LA (biomarker)	-	-	[‡]0.44	-	-	-	-	-	-41.6%
Laursen *et al.* 2019	Prospective cohort	Q2	178-item semi-quantitative	Y, specific ρ	Substitutions between dairy products	Ocké *et al.* [illegible]	63 males + 58 females; 12 x [illegible]	Energy	0.71	0.58	-	-	-	-	2.7%	6.7%	-

Ref, Country	Study design	SCI-Q	FFQ details	Validation metrics reported in manuscript?	†Substitution examined	Validity data: Ref	*n* in validation study and reference method	Variable	^Unadjusted r or ρ			^Adjusted r or ρ			Difference from reference method		
									Male	Female	Total	Male	Female	Total	Male	Female	Total
Stuber *et al.* 2021	Prospective cohort	Q3		Y, specific ρ		1997b	24HR	Protein	0.61	0.51	-	0.71	0.67	-	2.1%	2.9%	-
Vissers *et al.* 2019	Prospective cohort	Q2		Y, specific ρ	FA from dairy substituted with FA from meat			CHO	0.72	0.66	-	0.74	0.76	-	1.0%	2.0%	-
Scheffers *et al.* 2022	Prospective cohort	Q2		Y, specific ρ for fruit	SSB or fruit with pure fruit juice			Fat	0.69	0.58	-	0.61	0.63	-	11.5%	14.1%	-
Liu *et al.* 2019	Prospective cohort	Q2		Y, range of ρ for total and individual SFA	Energy intake from total SFA with an equal amount of energy from each of the other macronutrients (PUFA, monounsaturated fatty acids (MUFA), trans fatty acids, total carbohydrates, animal protein, and vegetable protein)	Praagman *et al*. 2016		SFA	0.35	0.64	-	0.55	0.5	-	0.7%	4.7%	-
								PUFA	0.53	0.44	-	0.52	0.22	-	26.2%*	53.2%*	-
								MUFA	0.67	0.63	-	0.66	0.58	-	8.9%*	14.3%*	-
								TFA	0.63	0.56	-	0.53	0.49	-	10.5%*	7.7%	-
						Ocké *et al.* 1997a		Milk and milk products	0.71	0.79	-	-	-	-	36.0%*	21.2%*	-
								Cheese	0.64	0.38	-	-	-	-	16.7%	0.0%	-
								Meat	0.47	0.70	-	-	-	-	-5.1%	-8.6%*	-
								Fruit	0.68	0.56	-	-	-	-	-	-	-
Alferink *et al.* 2019	Prospective cohort	Q1	389-item semi-quantitative	N	Macronutrients with each other	Goldbohm *et al.* 1994	59 males + 48 females; 9d FR	Energy	-	-	‡0.74	-	-	-	-	-	14.5%
								Protein	-	-	‡0.61	-	-	‡0.59	-	-	11.5%
								CHO	-	-	‡0.77	-	-	‡0.71	-	-	-11.7%
								Fat	-	-	‡0.72	-	-	‡0.52	-	-	-17.7%
								SFA	-	-	‡0.73	-	-	‡0.58	-	-	-23.2%
								PUFA	-	-	‡0.73	-	-	‡0.75	-	-	4.7%
								Fibre	-	-	‡0.74	-	-	‡0.74	-	-	6.2%
Slurink *et al.* 2022	Prospective cohort	Q2	92 (HS1) to 104-item (HS2) semi-quantitative FFQ	Y, specific *r* for animal protein and SFA	High fat cheese with alternative dairy products	Grootenhuis *et al.* 1995 (HS1)	33 males + 42 females; DHI	Energy	-	-	‡0.72	-	-	-	-	-	0.0%
								Protein	-	-	‡0.69	-	-	-	-	-	1.4%
								CHO	-	-	‡0.72	-	-	-	-	-	-5.2%
								Fat	-	-	‡0.70	-	-	-	-	-	5.6%
								SFA	-	-	‡0.73	-	-	-	-	-	5.6%
								MUFA	-	-	‡0.72	-	-	-	-	-	6.1%
								PUFA	-	-	‡0.68	-	-	-	-	-	11.8%
								Fibre	-	-	‡0.70	-	-	-	-	-	13.0%
						Streppel *et al.* 2013 (HS2)	62 males + 66 females; 3 x 24HR	Energy	-	-	‡0.55	-	-	-	-	-	1.2%
								Protein	-	-	‡0.51	-	-	‡0.50	-	-	-5.3%
								CHO	-	-	‡0.70	-	-	‡0.66	-	-	-1.4%
								Fat	-	-	‡0.39	-	-	‡0.43	-	-	8.6%
								SFA	-	-	‡0.44	-	-	‡0.57	-	-	7.7%
								MUFA	-	-	‡0.33	-	-	‡0.33	-	-	13.0%
								PUFA	-	-	‡0.44	-	-	‡0.44	-	-	14.3%
								TFA	-	-	‡0.42	-	-	‡0.44	-	-	0.0%
								Cheese	-	-	‡0.46	-	-	‡0.51	-	-	20.0%
								Milk and milk products	-	-	‡0.69	-	-	‡0.68	-	-	-5.2%
Buso *et al.* 2023	Prospective cohort with 5 separate cohorts (Lifelines, Nqplus, AOC, PREDIMED-Plus, Feel4Diabetes)	Q2	110-item semi-quantitative (Lifelines)	N	SSB with LNCB or SSB/LNCB with water	Brouwer-Brolsma *et al.* 2022	191 males + 210 females; a validated regular FFQ	SSB	-	-	0.58	-	-	-	-	-	35.0%
								LNCB	-	-	0.47	-	-	-	-	-	17.4%
								Water	-	-	0.07	-	-	-	-	-	2153.0%
								Fruit juice	-	-	0.65	-	-	-	-	-	-6.0%
			183-item semi-quantitative (Nqplus)			Siebelink *et al.* 2011	174 males + 342 females	Energy	‡0.74	‡0.80	‡0.82	-	-	-	-4.5%	0.0%	-1.8%
						Feunekes *et al.* 1993	95 males + 96 females; DHI + erythrocyte membrane FA (biomarker)	Energy	‡0.77	‡0.76	‡0.83	-	-	-	-	-	3.4%
								Fat	‡0.71	‡0.74	‡0.78	-	-	-	-	-	10.8%
								SFA	‡0.68	‡0.74	‡0.75	-	-	-	-	-	9.3%
								MUFA	‡0.71	‡0.75	‡0.78	-	-	-	-	-	8.6%
								PUFA	‡0.53	‡0.58	‡0.61	-	-	-	-	-	19.6%
								LA	‡0.48	‡0.53	‡0.56	-	-	-	-	-	22.4%
								PUFA (biomarker)	-	-	‡0.33	-	-	-	-	-	76.9%
								LA (biomarker)	-	-	‡0.44	-	-	-	-	-	-41.6%
						Streppel *et al.* 2013	62 males + 66 females; 3 x 24HR	Energy	-	-	‡0.55	-	-	-	-	-	1.2%
								Protein	-	-	‡0.51	-	-	‡0.50	-	-	-5.3%
								CHO	-	-	‡0.70	-	-	‡0.66	-	-	-1.4%
								Fat	-	-	‡0.39	-	-	‡0.43	-	-	8.6%
								SFA	-	-	‡0.44	-	-	‡0.57	-	-	7.7%
								MUFA	-	-	‡0.33	-	-	‡0.33	-	-	13.0%
								PUFA	-	-	‡0.44	-	-	‡0.44	-	-	14.3%
								TFA	-	-	‡0.42	-	-	‡0.44	-	-	0.0%
								Cheese	-	-	‡0.46	-	-	‡0.51	-	-	20.0%
								Milk and milk products	-	-	‡0.69	-	-	‡0.68	-	-	-5.2%
			203-item semi-quantitative (AOC)			Feunekes *et al.* 1993	95 males + 96 females; DHI + erythrocyte membrane FA (biomarker)	Energy	‡0.77	‡0.76	‡0.83	-	-	-	-	-	3.4%
								Fat	‡0.71	‡0.74	‡0.78	-	-	-	-	-	10.8%
								SFA	‡0.68	‡0.74	‡0.75	-	-	-	-	-	9.3%
								MUFA	‡0.71	‡0.75	‡0.78	-	-	-	-	-	8.6%
								PUFA	‡0.53	‡0.58	‡0.61	-	-	-	-	-	19.6%
								LA	‡0.48	‡0.53	‡0.56	-	-	-	-	-	22.4%
								PUFA (biomarker)	-	-	‡0.33	-	-	-	-	-	76.9%
								LA (biomarker)	-	-	‡0.44	-	-	-	-	-	-41.6%
			143-item semi-quantitative (PREDIMED-PLUS)			Fernańdez-Ballart *et al.* 2010	73 males + 85 females; 4 x 3d FR	Energy	-	-	‡0.36	-	-	-	-	-	11.0%
								Protein	-	‡0.52	‡0.28	-	‡0.44	‡0.40	-	-5.7%	6.7%
								CHO	-	‡0.44	‡0.38	-	‡0.42	‡0.56	-	-2.6%	5.5%
			33-item semi-quantitative (Feel4Diabetes)			None cited	No information provided	None provided	-	-	-	-	-	-	-	-	-
Norway																	
Haugsgjerd *et al.* 2022	Prospective cohort	Q2	169-item semi-quantitative	Y, specific ρ	CHO with SFA	Anderson *et al.* 1999 (male) Nes *et al.* 1992 (female)	125 males; 14d WFR 38 females; 14d WFR	Energy	0.48	0.40	-	-	-	-	-5.5%	-2.6%	-
Bratveit *et al.* 2024	Cross-sectional	Q2		N	Macronutrients with each other			Protein	0.44	0.65	-	-	0.66	-	2.1%	5.6%	-
								CHO	0.57	0.50	-	-	0.76	-	0.3%*	-0.9%	-
								Fat	0.46	0.55	-	-	0.68	-	-13.6%	-11.0%	-
								SFA	0.44	0.60	-	-	0.79	-	-15.3%*	-13.3%	-
								MUFA	0.45	0.59	-	-	0.61	-	-12.8%*	-12.0%	-
								PUFA	0.52	0.56	-	-	0.57	-	-5.7%	0.0%	-
Arnesen *et al.* 2024	Prospective cohort	Q2	61-item semi-quantitative	Y, qualitative statement only	Replacement of SFA with other macronutrients	Solvoll 1983	Information from the citation is unavailable online	-	-	-	-	-	-	-	-	-	-
Singapore																	
Jiang *et al.* 2020a	Prospective cohort	Q2	165-item semi-quantitative	Y, range of ρ	PUFA/MUFA for SFA or total CHO	Hankin *et al.* 2001	332 males + 478 females; 2 x 24HR	Energy (Cantonese / Hokkien)	0.32 / 0.53	0.31 / 0.51	-	-	-	-	-	-	1.7%
Jiang *et al.* 2020b	Prospective cohort	Q2		Y, range of ρ for protein and fat only	Meat with a different type of protein (red meat, poultry, and fish)			Protein (Cantonese / Hokkien)	0.36 / 0.46	0.36 / 0.46	-	0.36 / 0.36	0.61 / 0.50	-	-	-	-4.5%
Ying *et al.* 2024	Prospective cohort	Q2		Y, range of ρ for protein and fat only				CHO (Cantonese / Hokkien)	0.37 / 0.58	0.32 / 0.56	-	0.40 / 0.56	0.48 / 0.50	-	-	-	9.9%
Seah *et al.* 2019	Prospective cohort	Q2		Y, specific ρ for CHO only	Rice for other foods (red meat, fish or poultry, fruit, soy, and bread)			Fat (Cantonese / Hokkien)	0.29 / 0.41	0.42 / 0.44	-	0.44 / 0.41	0.47 / 0.34	-	-	-	-10.2%
								SFA (Cantonese / Hokkien)	0.42 / 0.52	0.42 / 0.41	-	0.73 / 0.52	0.52 / 0.24	-	-	-	-8.3%
Lee *et al.* 2023	Prospective cohort	Q2	163-item semi-quantitative	Y, range of r	Total plant-based proteins for animal-based protein food groups	Whitton *et al.* 2017	81 males + 80 females; 2 x 24HR (nutrients) and biomarkers (food group)	Energy	-	-	‡0.15	-	-	‡0.04	-	-	-
								Protein (%E)	-	-	‡0.39	-	-	‡0.53	-	-	-
								Soy protein	-	-	‡0.21	-	-	‡0.27	-	-	-
								Fish and seafood	-	-	‡0.47	-	-	‡0.51	-	-	-
Lim *et al.* 2021	Prospective cohort	Q2	169-item semi-quantitative	Y, range of r	Protein or fat for CHO	Deurenberg-Yap *et al.* 2000	42 males + 84 females; 3 x 24HR	Energy	-	-	‡0.56	-	-	-	-	-	3.6%
								Protein	-	-	‡0.46	-	-	-	-	-	7.6%
								Fat	-	-	‡0.58	-	-	-	-	-	2.7%
								SFA	-	-	‡0.51	-	-	-	-	-	2.2%
								MUFA	-	-	‡0.50	-	-	-	-	-	0.9%
								PUFA	-	-	‡0.39	-	-	-	-	-	10.1%
South Korea																	
Chung *et al.* 2023	Prospective cohort	Q1	103-item semi-quantitative	N	Fish for red and processed meat or processed fish	Ahn *et al.* 2007	35 males + 89 females; 12d FR	Energy	-	-	0.33	-	-	-	-	-	10.7%
								Protein	-	-	0.28	-	-	0.34	-	-	0.1%
								CHO	-	-	0.27	-	-	0.49	-	-	20.3%
								Fat	-	-	0.31	-	-	0.45	-	-	-20.4%
Spain																	
Santiago *et al.* 2018	Prospective cohort	Q1	136-item semi-quantitative	N	MUFA or PUFA for TFA or SFA; CHO for SFA	Fernańdez-Ballart *et al.* 2010	73 males + 85 females; 4 x 3d FR	Energy	-	-	‡0.36	-	-	-	-	-	11.0%
Dominguez *et al.* 2018	Prospective cohort	Q1		N	MUFA, PUFA, CHO by SFA; Fish, potatoes, poultry, eggs, vegetables, fruits and nuts, and cereals by red meat			Protein	-	‡0.52	‡0.28	-	‡0.44	‡0.40	-	-5.7%	6.7%
								CHO	-	‡0.44	‡0.38	-	‡0.42	‡0.56	-	-2.6%	5.5%
Beulen *et al.* 2018	Prospective cohort	Q1		N	Macronutrients for each other; high-fat food for higher-quality options (meat, fish and seafood; oils, butter and margarine; and nuts)	Martin-Moreno *et al*. 1993	147 females; 4 x 4d FR	Fat	-	‡0.41	‡0.47	-	‡0.41	‡0.46	-	-1.5%	9.3%
								SFA	-	‡0.47	‡0.52	-	‡0.40	‡0.61	-	-5.5%	33.0%
								MUFA	-	‡0.40	‡0.51	-	‡0.35	‡0.44	-	-3.0%	4.9%
								PUFA	-	‡0.45	‡0.43	-	‡0.37	‡0.42	-	-14.2%	18.1%
								Fibre	-	‡0.51	‡0.49	-	‡0.51	‡0.60	-	-3.3%	20.9%
								Fish or seafood	-	-	‡0.42	-	-	-	-	-	12.4%
								Potatoes	-	-	‡0.37	-	-	-	-	-	-0.9%

Ref, Country	Study design	SCI-Q	FFQ details	Validation metrics reported in manuscript?	[†]Substitution examined	Validity data											
						Ref	*n* in validation study and reference method	Variable	^Unadjusted r or ρ			^Adjusted r or ρ			Difference from reference method		
									Male	Female	Total	Male	Female	Total	Male	Female	Total
								Meat or meat products	-	-	‡0.61	-	-	-	-	-	18.4%
								Eggs	-	-	‡0.42	-	-	-	-	-	-15.7%
								Fruits	-	-	‡0.57	-	-	-	-	-	-2.4%
								Total nuts	-	-	‡0.38	-	-	-	-	-	61.9%
								Olive oil	-	-	‡0.55	-	-	-	-	-	-22.4%
								Dairy products	-	-	‡0.81	-	-	-	-	-	-33.8%
								Cereals	-	-	‡0.58	-	-	-	-	-	-3.0%
Dierssen-Sotos *et al.* 2020	Case-control	Q2	140-item semi-quantitative	N	Fat subtypes for each other	Not validated	No information provided	None provided	-	-	-	-	-	-	-	-	-
Sweden																	
Hantikainen *et al.* 2022	Prospective cohort	Q1	85-item semi-quantitative	N	Dietary fat sub-types with each other	Messerer *et al.* 2004	248 males; 14 x 24HR	Energy	-	-	-	-	-	-	-7.6%	-	-
								Protein	-	-	-	0.44	-	-	6.0%	-	-
								CHO	-	-	-	0.73	-	-	-10.2%	-	-
								Fat	-	-	-	0.70	-	-	-7.4%	-	-
								SFA	-	-	-	0.75	-	-	-2.1%	-	-
								PUFA	-	-	-	0.49	-	-	-8.6%	-	-
								MUFA	-	-	-	0.66	-	-	-13.1%	-	-
Switzerland																	
Ortega *et al.* 2024	Prospective cohort	Q1	97-item semi-quantitative FFQ	N	Dairy for meat, fish, eggs, fruits and vegetables	Bernstein *et al.* 1995	Information from the citation is unavailable online	None reported	-	-	-	-	-	-	-	-	-
UK																	
Wang *et al.* 2022	Prospective cohort	Q2	Inadequate details provided	N	Poultry or cereal for red meat	None cited	No information provided	None reported	-	-	-	-	-	-	-	-	-
US																	
Lo *et al.* 2020	Prospective cohort	Q1	110 item quantitative	N	Meat with each other	Block *et al.* 1986	17 males + 33 females; 1 x 24HR	Energy	-	-	‡0.82	-	-	-	-	-	-
Anderson *et al.* 2018	Case-control	Q1		N	Macronutrients for each other			Fat	-	-	‡0.73	-	-	-	-	-	-
Su *et al.* 2022	Cross-sectional	-	140-item quantitative	N	Fish for red and processed meat, whole grain for refined grain, and whole fruit for fruit juice	Patterson *et al.* 1999	113 females; 4 x 24HR + 1 x 4d FR	Energy	-	‡0.37	-	-	-	-	-	-6.6%	-
								Protein	-	‡0.36	-	-	‡0.51	-	-	-1.5%	-
								CHO	-	‡0.41	-	-	‡0.67	-	-	-13.0%	-
								Fat	-	‡0.49	-	-	‡0.64	-	-	0.0%	-
								SFA	-	‡0.44	-	-	‡0.63	-	-	12.5%	-
Huang *et al.* 2020	Prospective cohort	Q1	124-item semi-quantitative	N	Plant protein for animal protein, egg protein or red meat protein	Thompson *et al.* 2008	987 males + 966 females; 2 x 24HR	Energy	‡0.39	‡0.22	-	-	-	-	-13.6%	-4.9%	-
Liao *et al.* 2019	Prospective cohort	Q3		N				Protein	‡0.41	‡0.30	-	‡0.43	‡0.30	-	-18.2%	-9.5%	-
Zhao *et al.* 2024	Prospective cohort	Q1		N	Plant fat with animal fat from red meat, white meat, dairy, egg or fish			CHO	‡0.39	‡0.32	-	‡0.71	‡0.64	-	-11.9%	-2.2%	-
								Fat	‡0.58	‡0.39	-	‡0.72	‡0.62	-	-13.9%	-4.5%	-
								SFA	‡0.65	‡0.48	-	‡0.76	‡0.69	-	-17.2%	-10.4%	-
								F&V	‡0.59	‡0.46	-	‡0.72	‡0.61	-	0.8%	16.2%	-
Guasch-Ferré *et al.* 2022	Prospective cohort	Q1	152-item semi-quantitative	N	Olive oil for margarine, butter, mayonnaise and dairy fat	Yuan *et al.* 2018	627 females; 2 x 7d FR + 4 x 24HR + biomarkers	Energy	-	0.12	-	-	-	-	-	-15.6%	-
Tessier *et al*. 2024	Prospective cohort	Q1		N				Protein	-	0.26	-	-	0.28	-	-	0.8%	-
Lee *et al.* 2022	Prospective cohort (NHS + HPFS)	Q1		Y, specific ρ	Plant proteins with animal protein sources (processed red meat, unprocessed red meat, poultry, fatty and non-fatty fish, egg, milk, yoghurt, cheese and other dairy)			SFA	-	0.12	-	-	0.10	-	-	13.4%	-
Guasch-Ferré *et al*. 2020	Prospective cohort	Q1		N	Olive oil for margarine, butter, mayonnaise, other plant oil, dairy fat, and all other fats combined			MUFA	-	0.04	-	-	0.06	-	-	71.2%	-
Korat *et al.* 2019	Prospective cohort	Q1		Y, specific ρ	Dairy fat with other macronutrients			PUFA	-	0.19	-	-	0.17	-	-	-50.4%	-
Schmid *et al.* 2020	Prospective cohort	Q1		Y, specific ρ	Yoghurt with red meat, processed meat, nuts, whole grains, cheese, milk or total other dairy foods			TFA	-	0.30	-	-	0.27	-	-	145.5%	-
Wan *et al.* 2022	Prospective cohort	Q2		N	Energy from specific types of fat with the equivalent calories from other nutrients	Al-Shaar *et al.* 2021	671 males; 2 x 7d FR	Energy	0.27	-	-	-	-	-	-9.7%	-	-
Wurtz et al. 2021b	Prospective cohort	Q1		Y, range of ρ	Red meat with another protein source			Protein	0.22	-	-	0.28	-	-	-10.5%	-	
Dennis et al. 2023	Prospective cohort	Q1		Y, specific ρ	Equivalent energy from sugars or starch for an equivalent percentage of energy from total fat; Energy from total glucose equivalent or total fructose equivalent for equivalent energy from vegetable and animal fat			CHO	0.55	-	-	0.75	-	-	-9.9%	-	-
								Sugars	0.64	-	-	0.70	-	-	-2.7%	-	-
								Fat	0.43	-	-	0.67	-	-	-10.2%	-	-
Nassan *et al.* 2018	Prospective cohort	Q1		Y, specific ρ for poultry and processed red meat	Meat, processed meat or other protein rich foods with fish			SFA	0.49	-	-	0.71	-	-	-14.3%	-	-
								PUFA	0.42	-	-	0.51	-	-	-14.3%	-	-
								MUFA	0.45	-	-	0.59	-	-	-3.1%	-	-
						Yuan *et al.* 2017	632 females; 2 x 7d FR	Energy	-	0.28	-	-	-	-	-	6.7%	-
								Protein	-	0.33	-	-	0.48	-	-	11.0%	-
								CHO	-	0.41	-	-	0.65	-	-	5.3%	-
								Sugars	-	0.53	-	-	0.68	-	-	8.7%	-
								Fat	-	0.36	-	-	0.59	-	-	4.5%	-
								SFA	-	0.44	-	-	0.61	-	-	0.0%	-
								PUFA	-	0.37	-	-	0.48	-	-	0.0%	-
								MUFA	-	0.28	-	-	0.47	-	-	12.5%	-
								Animal protein	-	0.42	-	-	0.56	-	-	10.7%	-
								Vegetable protein	-	0.39	-	-	0.66	-	-	11.7%	-
						Song *et al.* 2016	624 females; 2 x 7d FR	Animal protein	-	-	-	-	0.56	-	-	-	-
								Plant protein	-	-	-	-	0.66	-	-	-	-
						Hu *et al.* 1999	127 males; 2 x 7d FR	Processed meat	‡0.52	-	-	-	-	-		-	-
								Meat / red meats	‡0.59	-	-	-	-	-		-	-
								Poultry	‡0.48	-	-	-	-	-		-	-
								Fish / seafood	‡0.74	-	-	-	-	-		-	-
								Low-fat dairy products	‡0.62	-	-	-	-	-		-	-
								High-fat dairy products	‡0.62	-	-	-	-	-		-	-
								Eggs	‡0.56	-	-	-	-	-		-	-
								Legumes	‡0.46	-	-	-	-	-		-	-
								Nuts	‡0.45	-	-	-	-	-		-	-
						Salvini *et al.* 1989	173 females; 4 x 7d FR	Skimmed milk	-	‡0.79	-	-	‡0.81	-	-	66.7%	-
								Whole milk	-	‡0.62	-	-	‡0.62	-	-	-33.3%	-
								Yoghurt	-	‡0.74	-	-	‡0.94	-	-	200.0%	-
								Ice cream	-	‡0.61	-	-	‡0.73	-	-	0.0%	-
								Cottage cheese	-	‡0.68	-	-	‡0.80	-	-	0.0%	-
								Hard cheese	-	‡0.49	-	-	‡0.57	-	-	0.0%	-
								Margarine	-	‡0.71	-	-	‡0.76	-	-	25.0%	-
								Butter	-	‡0.79	-	-	‡0.85	-	-	-40%	-
						Feskanich *et al.* 1993	157 males; 2 x 7d FR	Low-fat/skimmed milk	0.82	-	-	0.88	-	-	33.3%	-	-
								Whole milk	0.59	-	-	0.67	-	-	-41.2%	-	-
								Yoghurt	0.76	-	-	0.86	-	-	100.0%	-	-
								Cottage/ricotta cheese	0.28	-	-	0.52	-	-	0.0%	-	-
								Cream cheese	0.51	-	-	0.75	-	-	11.1%	-	-
								Other cheese	0.41	-	-	0.56	-	-	-43.8%	-	-
								Eggs	0.57	-	-	0.76	-	-	-26.7%	-	-
								Chicken/turkey with skin	0.49	-	-	-	-	-	83.3%	-	-
								Chicken/turkey without skin	0.30	-	-	0.56	-	-	-3.6%	-	-
								Processed meats	0.55	-	-	0.83	-	-	-70.3%	-	-
								Beef/pork/lamb	0.45	-	-	0.66	-	-	-21.1%	-	-
								Canned tuna	0.56	-	-	0.73	-	-	57.1%	-	-
								Dark meat fish	0.42	-	-	0.58	-	-	-18.2%	-	-
								Other fish	0.39	-	-	-	-	-	6.3%	-	-
								Shrimp/lobster/scallops	0.23	-	-	-	-	-	-40.0%	-	-
						Rimm *et al.* 1992	127 males; 2 x 7d FR	Energy	‡0.40	-	-	-	-	-	-7.1%	-	-
								Protein	‡0.25	-	-	‡0.38	-	-	-7.7%	-	-
								CHO	‡0.48	-	-	‡0.69	-	-	-2.1%	-	-
								Fat	‡0.52	-	-	‡0.61	-	-	-14.9%	-	-
								SFA	‡0.63	-	-	‡0.71	-	-	-14.2%	-	-
								MUFA	‡0.53	-	-	‡0.62	-	-	-14.1%	-	-
								PUFA	‡0.33	-	-	‡0.29	-	-	-18.3%	-	-
								Fibre	‡0.49	-	-	‡0.64	-	-	12.5%	-	-
Wesselink *et al.* 2022	Prospective cohort	Q2	152-item semi-quantitative	Y, range of *r* or ρ	Substituting a particular type of protein for fats and carbohydrates<br>Protein-rich food at the expense of another	Subar *et al.* 2001	403 males + females; 4 x 24HR	Energy	0.49	0.48	-	-	-	-	-	-	-
								Protein	0.47	0.46	-	0.57	0.60	-	-	-	-
								CHO	0.53	0.50	-	0.63	0.69	-	-	-	-
								Fat	0.52	0.55	-	0.62	0.66	-	-	-	-
								SFA	0.57	0.60	-	0.68	0.66	-	-	-	-
								MUFA	0.51	0.56	-	0.60	0.62	-	-	-	-
								PUFA	0.52	0.48	-	0.61	0.64	-	-	-	-
						Millen *et al.* 2005	436 males + 497 females; 4 x 24HR	Meat / red meat	0.53	0.48	-	0.61	0.58	-	-15.2%	-7.6%	-
								Total dairy	0.73	0.76	-	0.80	0.78	-	0.6%	11.9%	-
								Grains	0.53	0.48	-	0.63	0.59	-	-20.8%	-16.1%	-

Ref, Country	Study design	SCI-Q	FFQ details	Validation metrics reported in manuscript?	†Substitution examined	Validity data: Ref	n in validation study and reference method	Variable	^Unadjusted r or ρ: Male	^Unadjusted r or ρ: Female	^Unadjusted r or ρ: Total	^Adjusted r or ρ: Male	^Adjusted r or ρ: Female	^Adjusted r or ρ: Total	Difference from reference method: Male	Difference from reference method: Female	Difference from reference method: Total
			220-item semi-quantitative			Knudsen *et al.* 2016	97 females; 1 x 4d FR	Energy	-	-	‡0.29	-	-	-	-	-	-9.5%
								Protein	-	-	‡0.49	-	-	-	-	-	-9.5%
								CHO	-	-	‡0.63	-	-	-	-	-	-8.2%
								Fat	-	-	‡0.56	-	-	-	-	-	-14.7%
								SFA	-	-	‡0.51	-	-	-	-	-	-9.9%
								MUFA	-	-	‡0.52	-	-	-	-	-	-17.5%
								PUFA	-	-	‡0.41	-	-	-	-	-	-19.0%
								Meat / red meat	-	-	‡0.41	-	-	-	-	-	-15.4%
								High fat dairy	-	-	‡0.42	-	-	-	-	-	69.2%
								Low fat dairy	-	-	‡0.61	-	-	-	-	-	73.6%
								Poultry	-	-	‡0.32	-	-	-	-	-	25.0%
								Eggs	-	-	‡0.47	-	-	-	-	-	-4.3%
								Fish / seafood	-	-	‡0.38	-	-	-	-	-	0.0%
Du *et al.* 2022	Prospective cohort	Q1	61-item semi-quantitative	N	Unprocessed/Minimally processed food (NOVA-1) and culinary ingredients (NOVA-2) for ultra-processed foods (NOVA-4)	Willett *et al.* 1985	225 females; 4 x 7d FR	Protein	-	‡0.33	-	-	‡0.47	-	-	4.7%	-
								CHO	-	‡0.53	-	-	‡0.45	-	-	-14.7%	-
								Fat	-	‡0.39	-	-	‡0.53	-	-	-18.2%	-
								SFA	-	‡0.44	-	-	‡0.59	-	-	-12.0%	-
								PUFA	-	‡0.40	-	-	‡0.48	-	-	-32.4%	-
								Fibre	-	‡0.46	-	-	‡0.58	-	-	8.0%	-
Yang *et al.* 2022b	Prospective cohort	Q1		Y, specific r	Tea, SSB, ASB or FJ with coffee	Salvini *et al.* 1989	173 females; 4 x 7d FR	Tea	-	‡0.75	-	-	‡0.93	-	-	42.9%	-
								Coffee	-	‡0.90	-	-	‡0.78	-	-	33.3%	-
								Coke/Pepsi	-	‡0.81	-	-	‡0.84	-	-	100.0%	-
								Non-cola carbonated beverages	-	‡0.28	-	-	‡0.36	-	-	0.0%	-
Zhu *et al.* 2019	Prospective cohort	Q1	Block FFQ; 94-items semi-quantitative	N	Empty calories with total fruit, whole fruits, total vegetables, greens and beans, whole grains, dairy, total protein foods, and seafood and plant proteins based on HEI-2010 point calculations	Block *et al.* 1990	260 females; 3 x 4d FR	Energy	-	0.51	-	-	-	-	-	-3.2%	-
								Protein	-	0.48	-	-	-	-	-	2.5%	-
								CHO	-	0.51	-	-	-	-	-	-7.7%	-
								Fat	-	0.6	-	-	-	-	-	-3.9%	-
								SFA	-	0.63	-	-	-	-	-	-6.1%	-
								MUFA	-	0.59	-	-	-	-	-	-2.6%	-
								PUFA	-	0.48	-	-	-	-	-	-5.8%	-
Lin *et al.* 2021	Cross-sectional	Q1	166-item semi-quantitative	N	White rice with other grains	None cited	No information provided	None provided	-	-	-	-	-	-	-	-	-
Tooze *et al.* 2020	Prospective cohort	Q1	85-item semi-quantitative	N	PUFAs from different food sources with each other	Liese *et al.* 2015	80 males + 77 females; 3 x 24HR	Energy	-	-	‡0.36	-	-	-	-	-	-16.6%
								Fat	-	-	‡0.40	-	-	‡0.41	-	-	-10.0%
								SFA	-	-	‡0.42	-	-	‡0.39	-	-	-9.3%
								Bread, cereal, rice and pasta	-	-	‡0.15	-	-	‡0.26	-	-	-
								Fruits and vegetables	-	-	‡0.22	-	-	‡0.41	-	-	-
								Dairy	-	-	‡0.47	-	-	‡0.63	-	-	-
								Meat (beef, pork, non-poultry lunch meat)	-	-	‡0.33	-	-	‡0.48	-	-	-
								Poultry	-	-	‡0.22	-	-	‡0.24	-	-	-
								Eggs	-	-	‡0.45	-	-	‡0.60	-	-	-
								Nuts and seeds	-	-	‡0.25	-	-	‡0.41	-	-	-
								Fats and oils	-	-	‡0.08	-	-	‡0.09	-	-	-
								Chips, crackers, popcorn, pretzels	-	-	‡0.19	-	-	‡-0.04	-	-	-

Footnotes

[†]**Red** font denotes substitution of non-validated variables; **amber** denotes some substituted variables were

[^]Spearman correlation coefficient displayed unless marked with [‡], which denotes Pearson correlation coefficient is displayed. The FFQs are considered to have poor (correlation < 0.2), fair (correlation between 0.2 – 0.4),

[*]Significantly different from reference methods. Values not marked with * were either not tested statistically, or

List of abbreviations used

Abbreviation	Full form
24HR	24-hour recall
AOC	Alpha Omega Cohort
CHO	Carbohydrate
DHI	Diet history interview
DLW	Doubly-labelled water
FA	Fatty acid
FFQ	Food frequency questionnaire
FR	Food record
HS1	Hoorn Study 1
HS2	Hoorn Study 2
LA	Linoleic acid
MUFA	Monounsaturated fat
N	No
PUFA	Polyunsaturated fat
Q1	Quartile 1
Q2	Quartile 2
Q3	Quartile 3
Q4	Quartile 4
SCI	Science Citation Index
SFA	Saturated fat
TFA	Trans-fat
WFR	Weighed food record
Y	Yes

References

Aghayan, M., G. Asghari, E. Yuzbashian, P. Dehghan, H. Khadem Haghighian, P. Mirmiran and M. Javadi (2019). "Association of nuts and unhealthy snacks with subclinical atherosclerosis among children and adolescents with overweight and obesity." Nutr Metab (Lond) **16**: 23.

Ahn, Y., E. Kwon, J. E. Shim, M. K. Park, Y. Joo, K. Kimm, C. Park and D. H. Kim (2007). "Validation and reproducibility of food frequency questionnaire for Korean genome epidemiologic study." European Journal of Clinical Nutrition **61**(12): 1435-1441.

Ahola, A. J., C. Forsblom and P. H. Groop (2018). "Association between depressive symptoms and dietary intake in patients with type 1 diabetes." Diabetes Res Clin Pract **139**: 91-99.

Ahola, A. J., M. I. Lassenius, C. Forsblom, V. Harjutsalo, M. Lehto and P. H. Groop (2017). "Dietary patterns reflecting healthy food choices are associated with lower serum LPS activity." Scientific Reports **7**(1): 6511.

Al-Shaar, L., C. Yuan, B. Rosner, S. B. Dean, K. L. Ivey, C. M. Clowry, L. A. Sampson, J. B. Barnett, J. Rood, L. J. Harnack, J. Block, J. E. Manson, M. J. Stampfer, W. C. Willett and E. B. Rimm (2021). "Reproducibility and Validity of a Semiquantitative Food Frequency Questionnaire in Men Assessed by Multiple Methods." American Journal of Epidemiology **190**(6): 1122-1132.

Alferink, L. J., J. C. Kiefte-de Jong, N. S. Erler, B. J. Veldt, J. D. Schoufour, R. J. de Knegt, M. A. Ikram, H. J. Metselaar, H. Janssen, O. H. Franco and S. Darwish Murad (2019). "Association of dietary macronutrient composition and non-alcoholic fatty liver disease in an ageing population: the Rotterdam Study." Gut **68**(6): 1088-1098.

Amirkalali, B., M. Khoonsari, M. R. Sohrabi, H. Ajdarkosh, N. Motamed, M. Maadi, H. Nobakht, E. Gholizadeh and F. Zamani (2021). "Relationship between dietary macronutrient composition and non-alcoholic fatty liver disease in lean and non-lean populations: a cross-sectional study." Public Health Nutr **24**(18): 6178-6190.

Andersen, L. F., K. Solvoll, L. R. Johansson, I. Salminen, A. Aro and C. A. Drevon (1999). "Evaluation of a food frequency questionnaire with weighed records, fatty acids, and alpha-tocopherol in adipose tissue and serum." Am J Epidemiol **150**(1): 75-87.

Anderson, C., Y. M. Mark Park, F. Z. Stanczyk, D. P. Sandler and H. B. Nichols (2018). "Dietary factors and serum antimullerian hormone concentrations in late premenopausal women." Fertility & Sterility **110**(6): 1145-1153.

Arnesen, E. K., I. Laake, M. B. Veierod and K. Retterstol (2024). "Saturated fatty acids and total and CVD mortality in Norway: a prospective cohort study with up to 45 years of follow-up." British Journal of Nutrition **132**(4): 466-478.

Bernstein, L., I. Huot and A. Morabia (1995). "Amélioration des performances d'un questionnaire alimentaire semi-quantitatif comparé à un rappel des 24 heures." Santé Publique **7**: 403-413.

Beulen, Y., M. A. Martínez-González, O. van de Rest, J. Salas-Salvadó, J. V. Sorlí, E. Gómez-Gracia, M. Fiol, R. Estruch, J. M. Santos-Lozano, H. Schröder, A. Alonso-Gómez, L. Serra-Majem, X. Pintó, E. Ros, N. Becerra-Tomas, J. I. González, M. Fitó, J. A. Martínez and A. Gea (2018). "Quality of Dietary Fat Intake and Body Weight and Obesity in a Mediterranean Population: Secondary Analyses within the PREDIMED Trial." Nutrients **10**(12): 2011.

Block, G., A. M. Hartman, C. M. Dresser, M. D. Carroll, J. Gannon and L. Gardner (1986). "A DATA-BASED APPROACH TO DIET QUESTIONNAIRE DESIGN AND TESTING." American Journal of Epidemiology **124**(3): 453-469.

Block, G., M. Woods, A. Potosky and C. Clifford (1990). "Validation of a self-administered diet history questionnaire using multiple diet records." J Clin Epidemiol **43**(12): 1327-1335.

Bratveit, M., A. Van Parys, T. Olsen, E. Strand, I. Marienborg, J. Laupsa-Borge, T. R. Haugsgjerd, A. McCann, I. Dhar, P. M. Ueland, J. Dierkes, S. N. Dankel, O. K. Nygard and V. Lysne (2024). "Association between dietary macronutrient composition and plasma one-carbon metabolites and B-vitamin cofactors in patients with stable angina pectoris." British Journal of Nutrition **131**(10): 1678-1690.

Brouwer-Brolsma, E. M., C. Perenboom, D. Sluik, A. van de Wiel, A. Geelen, E. J. M. Feskens and J. H. M. de Vries (2022). "Development and external validation of the 'Flower-FFQ': a FFQ designed for the Lifelines Cohort Study." Public Health Nutrition **25**(2): 225-236.

Budhathoki, S., N. Sawada, M. Iwasaki, T. Yamaji, A. Goto, A. Kotemori, J. Ishihara, R. Takachi, H. Charvat, T. Mizoue, H. Iso and S. Tsugane (2019). "Association of Animal and Plant Protein Intake With All-Cause and Cause-Specific Mortality in a Japanese Cohort." JAMA Intern Med **179**(11): 1509-1518.

Buso, M. E. C., E. M. Brouwer-Brolsma, N. D. Naomi, J. A. Harrold, J. C. G. Halford, A. Raben and E. J. M. Feskens (2022). "Dose-Response and Substitution Analyzes of Sweet Beverage Consumption and Body Weight in Dutch Adults: The Lifelines Cohort Study." Front Nutr **9**: 889042.

Buso, M. E. C., E. M. Brouwer-Brolsma, N. D. Naomi, J. Ngo, S. S. Soedamah-Muthu, C. Mavrogianni, J. A. Harrold, J. C. G. Halford, A. Raben, J. M. Geleijnse, Y. Manios, L. Serra-Majem and E. J. M. Feskens (2023). "Sugar and low/no-calorie-sweetened beverage consumption and associations with body weight and waist circumference changes in five European cohort studies: the SWEET project." European Journal of Nutrition **62**(7): 2905-2918.

Cheng, Y., H. Yan, M. J. Dibley, Y. Shen, Q. Li and L. Zeng (2008). "Validity and reproducibility of a semi-quantitative food frequency questionnaire for use among pregnant women in rural China." Asia Pac J Clin Nutr **17**(1): 166-177.

Chiu, T. H., H. Y. Huang, K. J. Chen, Y. R. Wu, J. P. Chiu, Y. H. Li, B. C. Chiu, C. L. Lin and M. N. Lin (2014). "Relative validity and reproducibility of a quantitative FFQ for assessing nutrient intakes of vegetarians in Taiwan." Public Health Nutr **17**(7): 1459-1466.
Chiu, T. H., M. N. Lin, W. H. Pan, Y. C. Chen and C. L. Lin (2018). "Vegetarian diet, food substitution, and nonalcoholic fatty liver." Ci Ji Yi Xue Za Zhi **30**(2): 102-109.
Chung, S., J. T. Hwang, H. Joung and S. Shin (2023). "Associations of Meat and Fish/Seafood Intake with All-Cause and Cause-Specific Mortality from Three Prospective Cohort Studies in Korea." Mol Nutr Food Res **67**(16): e2200900.
Date, C., M. Fukui, A. Yamamoto, K. Wakai, A. Ozeki, Y. Motohashi, C. Adachi, N. Okamoto, M. Kurosawa, Y. Tokudome, Y. Kurisu, Y. Watanabe, K. Ozasa, S. Nakagawa, N. Tokui, T. Yoshimura and A. Tamakoshi (2005). "Reproducibility and Validity of a Self-administered Food Frequency Questionnaire Used in the JACC Study." Journal of Epidemiology **15**(Supplement_I): S9-S23.
Dennis, K. K., F. Wang, Y. Li, J. E. Manson, E. B. Rimm, F. B. Hu, W. C. Willett, M. J. Stampfer and D. D. Wang (2023). "Associations of Dietary Sugar Types with Coronary Heart Disease Risk: A Prospective Cohort Study." Am J Clin Nutr **118**(5): 1005-1009.
Deurenberg-Yap, M., T. Li, W. L. Tan, W. A. van Staveren and P. Deurenberg (2000). "Validation of a semiquantitative food frequency questionnaire for estimation of intakes of energy, fats and cholesterol among Singaporeans." Asia Pac J Clin Nutr **9**(4): 282-288.
Dierssen-Sotos, T., I. Gómez-Acebo, C. Palazuelos, E. Gracia-Lavedan, B. Pérez-Gómez, M. Oribe, V. Martín, M. Guevara, P. Rodríguez-Cundín, G. Fernández-Tardón, R. Marcos-Gragera, A. Molina-Barceló, M. Díaz-Santos, G. Castaño-Vinyals, N. Aragonés, A. López-Gonzalez, P. Amiano, J. Castilla, J. Alonso-Molero and M. Kogevinas (2020). "Fatty acid intake and breast cancer in the Spanish multicase–control study on cancer (MCC-Spain)." European Journal of Nutrition **59**(3): 1171-1179.
Dominguez, L. J., M. Bes-Rastrollo, F. J. Basterra-Gortari, A. Gea, M. Barbagallo and M. A. Martinez-Gonzalez (2018). "Should we recommend reductions in saturated fat intake or in red/processed meat consumption? The SUN prospective cohort study." Clinical Nutrition **37**(4): 1389-1398.
Du, S., H. Kim, D. C. Crews, K. White and C. M. Rebholz (2022). "Association Between Ultraprocessed Food Consumption and Risk of Incident CKD: A Prospective Cohort Study." American Journal of Kidney Diseases **80**(5): 589-598.e581.
Esfahani, F. H., G. Asghari, P. Mirmiran and F. Azizi (2010). "Reproducibility and relative validity of food group intake in a food frequency questionnaire developed for the Tehran Lipid and Glucose Study." Journal of Epidemiology **20**(2): 150-158.
Esfandiar, Z., F. Hosseini-Esfahani, P. Mirmiran and F. Azizi (2021). "The association of dietary macronutrients composition with the incidence of type 2 diabetes, using iso-energetic substitution models: Tehran Lipid and Glucose Study." Prim Care Diabetes **15**(6): 1080-1085.
Fan, Y., Z. Li, J. Shi, S. Liu, L. Li, L. Ding, J. Zhao, Y. Pan, H. Lei, T. He, W. Li, X. Li, Y. Mi and L. Ma (2024). "The association between prepregnancy dietary fatty acids and risk of gestational diabetes mellitus: A prospective cohort study." Clinical Nutrition **43**(2): 484-493.
Fernández-Ballart, J. D., J. L. Piñol, I. Zazpe, D. Corella, P. Carrasco, E. Toledo, M. Perez-Bauer, M. A. Martínez-González, J. Salas-Salvadó and J. M. Martn-Moreno (2010). "Relative validity of a semi-quantitative food-frequency questionnaire in an elderly Mediterranean population of Spain." British Journal of Nutrition **103**(12): 1808-1816.
Ferreira, N. V., N. Gomes Goncalves, N. Khandpur, E. M. Steele, R. B. Levy, C. Monteiro, A. Goulart, A. R. Brunoni, P. Bacchi, P. Lotufo, I. Bensenor and C. K. Suemoto (2024). "Higher Ultraprocessed Food Consumption Is Associated With Depression Persistence and Higher Risk of Depression Incidence in the Brazilian Longitudinal Study of Adult Health." Journal of the Academy of Nutrition & Dietetics **18**: 18.
Feskanich, D., E. B. Rimm, E. L. Giovannucci, G. A. Colditz, M. J. Stampfer, L. B. Litin and W. C. Willett (1993). "Reproducibility and validity of food intake measurements from a semiquantitative food frequency questionnaire." J Am Diet Assoc **93**(7): 790-796.
Feunekes, G. I., W. A. Van Staveren, J. H. De Vries, J. Burema and J. G. Hautvast (1993). "Relative and biomarker-based validity of a food-frequency questionnaire estimating intake of fats and cholesterol." The American Journal of Clinical Nutrition **58**(4): 489-496.
Forootani, B., B. Sasanfar, A. Salehi-Abargouei and M. Mirzaei (2024). "The association between plant and animal protein intake with depression, anxiety, and stress." Nutritional Neuroscience **[Epub ahead of print]**: 1-14.
Gaeini, Z., P. Mirmiran, Z. Bahadoran, M. Aghayan and F. Azizi (2021). "The association between dietary fats and the incidence risk of cardiovascular outcomes: Tehran Lipid and Glucose Study." Nutr Metab (Lond) **18**(1): 96.
Goldbohm, R. A., P. A. van den Brandt, H. A. Brants, P. van't Veer, M. Al, F. Sturmans and R. J. Hermus (1994). "Validation of a dietary questionnaire used in a large-scale prospective cohort study on diet and cancer." Eur J Clin Nutr **48**(4): 253-265.
Grootenhuis, P. A., S. Westenbrink, C. M. Sie, J. N. de Neeling, F. J. Kok and L. M. Bouter (1995). "A semiquantitative food frequency questionnaire for use in epidemiologic research among the elderly: validation by comparison with dietary history." J Clin Epidemiol **48**(7): 859-868.

Guasch-Ferré, M., Y. Li, W. C. Willett, Q. Sun, L. Sampson, J. Salas-Salvadó, M. A. Martínez-González, M. J. Stampfer and F. B. Hu (2022). "Consumption of Olive Oil and Risk of Total and Cause-Specific Mortality Among U.S. Adults." J Am Coll Cardiol **79**(2): 101-112.

Guasch-Ferré, M., G. Liu, Y. Li, L. Sampson, J. E. Manson, J. Salas-Salvadó, M. A. Martínez-González, M. J. Stampfer, W. C. Willett, Q. Sun and F. B. Hu (2020). "Olive Oil Consumption and Cardiovascular Risk in U.S. Adults." Journal of the American College of Cardiology (JACC) **75**(15): 1729-1739.

Hankin, J. H., D. O. Stram, K. Arakawa, S. Park, S.-H. Low, H.-P. Lee and M. C. Yu (2001). "Singapore Chinese Health Study: Development, Validation, and Calibration of the Quantitative Food Frequency Questionnaire." Nutrition and Cancer **39**(2): 187-195.

Hansen, M. D., A. M. L. Würtz, C. P. Hansen, A. Tjønneland, E. B. Rimm, S. P. Johnsen, E. B. Schmidt, K. Overvad and M. U. Jakobsen (2021). "Substitutions between potatoes and other vegetables and risk of ischemic stroke." Eur J Nutr **60**(1): 229-237.

Hantikainen, E., E. Roos, R. Bellocco, A. D'Antonio, A. Grotta, H. O. Adami, W. Ye, Y. Trolle Lagerros and S. Bonn (2022). "Dietary fat intake and risk of Parkinson disease: results from the Swedish National March Cohort." Eur J Epidemiol **37**(6): 603-613.

Harris, C. P., A. von Berg, D. Berdel, C.-P. Bauer, T. Schikowski, S. Koletzko, J. Heinrich, H. Schulz and M. Standl (2018). "Association of Dietary Fatty Acids with Blood Lipids is Modified by Physical Activity in Adolescents: Results from the GINIplus and LISA Birth Cohort Studies." Nutrients **10**(10): 1372.

Haugsgjerd, T. R., G. M. Egeland, O. K. Nygård, J. Igland, G. Sulo, V. Lysne, K. J. Vinknes, K. Bjornevik and G. S. Tell (2022). "Intake of carbohydrates and SFA and risk of CHD in middle-age adults: the Hordaland Health Study (HUSK)." Public Health Nutr **25**(3): 634-648.

Hernández-Avila, M., I. Romieu, S. Parra, J. Hernández-Avila, H. Madrigal and W. Willett (1998). "Validity and reproducibility of a food frequency questionnaire to assess dietary intake of women living in Mexico City." Salud Publica Mex **40**(2): 133-140.

Hodge, A., A. J. Patterson, W. J. Brown, P. Ireland and G. Giles (2000). "The Anti Cancer Council of Victoria FFQ: relative validity of nutrient intakes compared with weighed food records in young to middle-aged women in a study of iron supplementation." Aust N Z J Public Health **24**(6): 576-583.

Hosseini-Esfahani, F., G. Koochakpoor, P. Mirmiran, S. Ebrahimof and F. Azizi (2019). "The association of dietary macronutrients with anthropometric changes, using iso-energetic substitution models: Tehran lipid and glucose study." Nutr Metab (Lond) **16**: 83.

Hosseini-Esfahani, F., G. Koochakpoor, Z. Tahmasebinejad, D. Khalili, P. Mirmiran and F. Azizi (2020). "The association of dietary macronutrients composition with the incidence of cardiovascular disease, using iso-energetic substitution models: Tehran lipid and glucose study." Nutr Metab Cardiovasc Dis **30**(12): 2186-2193.

Hu, F. B., E. Rimm, S. A. Smith-Warner, D. Feskanich, M. J. Stampfer, A. Ascherio, L. Sampson and W. C. Willett (1999). "Reproducibility and validity of dietary patterns assessed with a food-frequency questionnaire." Am J Clin Nutr **69**(2): 243-249.

Huang, J., L. M. Liao, S. J. Weinstein, R. Sinha, B. I. Graubard and D. Albanes (2020). "Association Between Plant and Animal Protein Intake and Overall and Cause-Specific Mortality." JAMA Intern Med **180**(9): 1173-1184.

Ibsen, D. B., K. Overvad, A. S. D. Laursen, J. Halkjær, A. Tjønneland, T. O. Kilpeläinen, E. T. Parner and M. U. Jakobsen (2021). "Changes in intake of dairy product subgroups and risk of type 2 diabetes: modelling specified food substitutions in the Danish Diet, Cancer and Health cohort." Eur J Nutr **60**(6): 3449-3459.

Ibsen, D. B., C. K. Warberg, A. M. L. Würtz, K. Overvad and C. C. Dahm (2019). "Substitution of red meat with poultry or fish and risk of type 2 diabetes: a Danish cohort study." Eur J Nutr **58**(7): 2705-2712.

Inan-Eroglu, E., O. Kuxhaus, F. Jannasch, D. V. Nickel and M. B. Schulze (2024). "Association between Protein Intake and Diabetes Complications Risk Following Incident Type 2 Diabetes: The EPIC-Potsdam Study." Metabolites **14**(3): 19.

Jahromi, M. K., H. Ahmadirad, H. Farhadnejad, M. Norouzzadeh, E. Mokhtari, F. Teymoori, N. Saber, Z. Heidari, P. Mirmiran and B. Rashidkhani (2024). "High-protein diet scores, macronutrient substitution, and breast cancer risk: insights from substitution analysis." BMC Women's Health **24**(1): 121.

Jiang, Y. W., L. T. Sheng, X. F. Pan, L. Feng, J. M. Yuan, A. Pan and W. P. Koh (2020b). "Meat consumption in midlife and risk of cognitive impairment in old age: the Singapore Chinese Health Study." Eur J Nutr **59**(4): 1729-1738.

Jiang, Y. W., L. T. Sheng, X. F. Pan, L. Feng, J. M. Yuan, A. Pan and W. P. Koh (2020a). "Midlife Dietary Intakes of Monounsaturated Acids, n-6 Polyunsaturated Acids, and Plant-Based Fat Are Inversely Associated with Risk of Cognitive Impairment in Older Singapore Chinese Adults." J Nutr **150**(4): 901-909.

Kabagambe, E. K., A. Baylin, D. A. Allan, X. Siles, D. Spiegelman and H. Campos (2001). "Application of the method of triads to evaluate the performance of food frequency questionnaires and biomarkers as indicators of long-term dietary intake." Am J Epidemiol **154**(12): 1126-1135.

Katsouyanni, K., E. B. Rimm, C. Gnardellis, D. Trichopoulos, E. Polychronopoulos and A. Trichopoulou (1997). "Reproducibility and relative validity of an extensive semi-quantitative food frequency questionnaire using dietary records and biochemical markers among Greek schoolteachers." Int J Epidemiol **26 Suppl 1**: S118-127.

Klipstein-Grobusch, K., J. H. den Breeijen, R. A. Goldbohm, J. M. Geleijnse, A. Hofman, D. E. Grobbee and J. C. M. Witteman (1998). "Dietary assessment in the elderly: validation of a semiquantitative food frequency questionnaire." European Journal of Clinical Nutrition **52**(8): 588-596.

Knudsen, V. K., E. E. Hatch, H. Cueto, K. L. Tucker, L. Wise, T. Christensen and E. M. Mikkelsen (2016). "Relative validity of a semi-quantitative, web-based FFQ used in the 'Snart Forældre' cohort – a Danish study of diet and fertility." Public Health Nutrition **19**(6): 1027-1034.
Korat, A. V. A., Y. Li, F. Sacks, B. Rosner, W. C. Willett, F. B. Hu and Q. Sun (2019). "Dairy fat intake and risk of type 2 diabetes in 3 cohorts of US men and women." American Journal of Clinical Nutrition **110**(5): 1192-1200.
Kouvari, M., T. Tsiampalis, R. I. Kosti, E. Damigou, C. Chrysohoou, G. Anastasiou, A. D. Koutsogianni, E. Liberopoulos, C. Tsioufis, P. P. Sfikakis, C. Pitsavos and D. Panagiotakos (2024). "The prolonged impact of swapping non-fermented with fermented dairy products on cardiovascular disease: the ATTICA cohort study (2002-2022)." European Journal of Clinical Nutrition **20**: 20.
Kroke, A., K. Klipstein-Grobusch, S. Voss, J. Möseneder, F. Thielecke, R. Noack and H. Boeing (1999). "Validation of a self-administered food-frequency questionnaire administered in the European Prospective Investigation into Cancer and Nutrition (EPIC) Study: comparison of energy, protein, and macronutrient intakes estimated with the doubly labeled water, urinary nitrogen, and repeated 24-h dietary recall methods." Am J Clin Nutr **70**(4): 439-447.
Kvist, K., A. S. D. Laursen, K. Overvad and M. U. Jakobsen (2020). "Substitution of Milk with Whole-Fat Yogurt Products or Cheese Is Associated with a Lower Risk of Myocardial Infarction: The Danish Diet, Cancer and Health cohort." J Nutr **150**(5): 1252-1258.
Lasota, A. N., M.-L. M. Grønholdt, C. S. Bork, S. Lundbye-Christensen, E. B. Schmidt and K. Overvad (2019). "Substitution of poultry and red meat with fish and the risk of peripheral arterial disease: a Danish cohort study." European Journal of Nutrition **58**(7): 2731-2739.
Laursen, A. S. D., C. C. Dahm, S. P. Johnsen, A. Tjønneland, K. Overvad and M. U. Jakobsen (2018). "Substitutions of dairy product intake and risk of stroke: a Danish cohort study." Eur J Epidemiol **33**(2): 201-212.
Laursen, A. S. D., I. Sluijs, J. M. A. Boer, W. M. M. Verschuren, Y. T. van der Schouw and M. U. Jakobsen (2019). "Substitutions between dairy products and risk of stroke: results from the European Investigation into Cancer and Nutrition-Netherlands (EPIC-NL) cohort." Br J Nutr **121**(12): 1398-1404.
Laursen, A. S. D., A. L. Thomsen, A. Beck, K. Overvad and M. U. Jakobsen (2022). "Theoretical substitutions between dairy products and all-cause and cause-specific mortality. Results from the Danish diet, cancer and health cohort." Br J Nutr **127**(10): 1557-1566.
Lee, D. H., F. K. Tabung and E. L. Giovannucci (2022). "Association of animal and plant protein intakes with biomarkers of insulin and insulin-like growth factor axis." Clinical Nutrition **41**(6): 1272-1280.
Lee, Y. Q., A. Chia, C. Whitton, D. Cameron-Smith, X. Sim, R. M. van Dam and F. F. C. M (2023). "Isocaloric Substitution of Plant-Based Protein for Animal-Based Protein and Cardiometabolic Risk Factors in a Multiethnic Asian Population." J Nutr **153**(5): 1555-1566.
Liao, L. M., E. Loftfield, A. Etemadi, B. I. Graubard and R. Sinha (2019). "Substitution of dietary protein sources in relation to colorectal cancer risk in the NIH-AARP cohort study." Cancer Causes Control **30**(10): 1127-1135.
Liese, A. D., J. L. Crandell, J. A. Tooze, M. T. Fangman, S. C. Couch, A. T. Merchant, R. A. Bell and E. J. Mayer-Davis (2015). "Relative validity and reliability of an FFQ in youth with type 1 diabetes." Public Health Nutr **18**(3): 428-437.
Lim, C. G. Y., C. Whitton, S. A. Rebello and R. M. van Dam (2021). "Diet Quality and Lower Refined Grain Consumption are Associated With Less Weight Gain in a Multi-Ethnic Asian Adult Population." Journal of Nutrition **151**(8): 2372-2382.
Lin, P. D., A. Cardenas, S. L. Rifas-Shiman, M. F. Hivert, T. James-Todd, C. Amarasiriwardena, R. O. Wright, M. L. Rahman and E. Oken (2021). "Diet and erythrocyte metal concentrations in early pregnancy-cross-sectional analysis in Project Viva." American Journal of Clinical Nutrition **114**(2): 540-549.
Liu, S., Y. T. van der Schouw, S. S. Soedamah-Muthu, A. M. W. Spijkerman and I. Sluijs (2019). "Intake of dietary saturated fatty acids and risk of type 2 diabetes in the European Prospective Investigation into Cancer and Nutrition-Netherlands cohort: associations by types, sources of fatty acids and substitution by macronutrients." Eur J Nutr **58**(3): 1125-1136.
Lo, J. J., Y. M. Park, R. Sinha and D. P. Sandler (2020). "Association between meat consumption and risk of breast cancer: Findings from the Sister Study." Int J Cancer **146**(8): 2156-2165.
Luan, D., D. Wang, H. Campos and A. Baylin (2020). "Red meat consumption and metabolic syndrome in the Costa Rica Heart Study." European Journal of Nutrition **59**(1): 185-193.
Lyskjaer, L., K. Overvad, A. Tjonneland and C. C. Dahm (2020). "Substitutions of Oatmeal and Breakfast Food Alternatives and the Rate of Stroke." Stroke **51**(1): 75-81.
MacDonald, C. J., A. L. Madkia, C. Mounier-Vehier, G. Severi and M. C. Boutron-Ruault (2023). "Associations between saturated fat intake and other dietary macronutrients and incident hypertension in a prospective study of French women." Eur J Nutr **62**(3): 1207-1215.
Martin-Moreno, J. M., P. Boyle, L. Gorgojo, P. Maisonneuve, J. C. Fernandez-Rodriguez, S. Salvini and W. C. Willett (1993). "Development and Validation of a Food Frequency Questionnaire in Spain." International Journal of Epidemiology **22**(3): 512-519.
Maukonen, M., K. Harald, N. E. Kaartinen, H. Tapanainen, D. Albanes, J. Eriksson, T. Härkänen, P. Jousilahti, S. Koskinen, E. Päivärinta, T. Suikki, H. Tolonen, A. M. Pajari and S. Männistö (2023). "Partial substitution of red or processed meat with plant-based foods and the risk of type 2 diabetes." Sci Rep **13**(1): 5874.

Melaku, Y. A., A. C. Reynolds, T. K. Gill, S. Appleton and R. Adams (2019). "Association between Macronutrient Intake and Excessive Daytime Sleepiness: An Iso-Caloric Substitution Analysis from the North West Adelaide Health Study." Nutrients **11**(10): 2374.

Messerer, M., A. Wolk and S.-E. Johansson (2004). "The Validity of Questionnaire-Based Micronutrient Intake Estimates Is Increased by Including Dietary Supplement Use in Swedish Men." The Journal of Nutrition **134**(7): 1800-1805.

Millen, A. E., D. Midthune, F. E. Thompson, V. Kipnis and A. F. Subar (2005). "The National Cancer Institute Diet History Questionnaire: Validation of Pyramid Food Servings." American Journal of Epidemiology **163**(3): 279-288.

Mirmiran, P., F. Hosseini Esfahani, Y. Mehrabi, M. Hedayati and F. Azizi (2010). "Reliability and relative validity of an FFQ for nutrients in the Tehran Lipid and Glucose Study." Public Health Nutrition **13**(5): 654-662.

Mirmiran, P., E. Yuzbashian, M. Aghayan, M. Mahdavi, G. Asghari and F. Azizi (2020). "A Prospective Study of Dietary Meat Intake and Risk of Incident Chronic Kidney Disease." J Ren Nutr **30**(2): 111-118.

Molina, M. d. C., I. M. Benseñor, O. Cardoso Lde, G. Velasquez-Melendez, M. Drehmer, T. S. Pereira, C. P. Faria, C. Melere, L. Manato, A. L. Gomes, J. Fonseca Mde and R. Sichieri (2013). "[Reproducibility and relative validity of the Food Frequency Questionnaire used in the ELSA-Brasil]." Cad Saude Publica **29**(2): 379-389.

Männistö, S., M. Virtanen, T. Mikkonen and P. Pietinen (1996). "Reproducibility and validity of a food frequency questionnaire in a case-control study on breast cancer." Journal of Clinical Epidemiology **49**(4): 401-409.

Morisaki, N., C. Nagata, S. Yasuo, S. Morokuma, K. Kato, M. Sanefuji, E. Shibata, M. Tsuji, A. Senju, T. Kawamoto, S. Ohga and K. Kusuhara (2018). "Optimal protein intake during pregnancy for reducing the risk of fetal growth restriction: the Japan Environment and Children's Study." British Journal of Nutrition **120**(12): 1432-1440.

Moslehi, N., Z. Kamali, P. Mirmiran, M. Barzin and A. Khalaj (2024). "Association of postoperative dietary macronutrient content and quality with total weight loss and fat-free mass loss at midterm after sleeve gastrectomy." Nutrition **120**: 112331.

Nanri, A., T. Mizoue, K. Kurotani, A. Goto, S. Oba, M. Noda, N. Sawada, S. Tsugane and G. for the Japan Public Health Center-Based Prospective Study (2015). "Low-Carbohydrate Diet and Type 2 Diabetes Risk in Japanese Men and Women: The Japan Public Health Center-Based Prospective Study." PLOS ONE **10**(2): e0118377.

Naomi, N. D., E. M. Brouwer-Brolsma, M. E. C. Buso, S. S. Soedamah-Muthu, J. A. Harrold, J. C. G. Halford, A. Raben, J. M. Geleijnse and E. J. M. Feskens (2023). "Association of sweetened beverages consumption with all-cause mortality risk among Dutch adults: the Lifelines Cohort Study (the SWEET project)." Eur J Nutr **62**(2): 797-806.

Nassan, F. L., Y. H. Chiu, J. C. Vanegas, A. J. Gaskins, P. L. Williams, J. B. Ford, J. Attaman, R. Hauser and J. E. Chavarro (2018). "Intake of protein-rich foods in relation to outcomes of infertility treatment with assisted reproductive technologies." Am J Clin Nutr **108**(5): 1104-1112.

Nes, M., L. Frost Andersen, K. Solvoll, B. Sandstad, B. E. Hustvedt, A. Løvø and C. A. Drevon (1992). "Accuracy of a quantitative food frequency questionnaire applied in elderly Norwegian women." Eur J Clin Nutr **46**(11): 809-821.

Nielsen, T. B., A. M. L. Würtz, A. Tjønneland, K. Overvad and C. C. Dahm (2022). "Substitution of unprocessed and processed red meat with poultry or fish and total and cause-specific mortality." Br J Nutr **127**(4): 563-569.

Ocké, M. C., H. B. Bueno-de-Mesquita, H. E. Goddijn, A. Jansen, M. A. Pols, W. A. van Staveren and D. Kromhout (1997a). "The Dutch EPIC food frequency questionnaire. I. Description of the questionnaire, and relative validity and reproducibility for food groups." Int J Epidemiol **26 Suppl 1**: S37-48.

Ocké, M. C., H. B. Bueno-de-Mesquita, M. A. Pols, H. A. Smit, W. A. van Staveren and D. Kromhout (1997b). "The Dutch EPIC food frequency questionnaire. II. Relative validity and reproducibility for nutrients." Int J Epidemiol **26 Suppl 1**: S49-58.

Okuda, M., K. Asakura and S. Sasaki (2019) "Protein Intake Estimated from Brief-Type Self-Administered Diet History Questionnaire and Urinary Urea Nitrogen Level in Adolescents." Nutrients **11**, 319 DOI: 10.3390/nu11020319.

Okuda, M. and S. Sasaki (2024). "Dietary Amino Acid Composition and Glycemic Biomarkers in Japanese Adolescents." Nutrients **16**(6): 19.

Oosterwijk, M. M., S. S. Soedamah-Muthu, J. M. Geleijnse, S. J. L. Bakker, G. Navis, S. H. Binnenmars, C. M. Gant and G. D. Laverman (2019). "High Dietary Intake of Vegetable Protein Is Associated With Lower Prevalence of Renal Function Impairment: Results of the Dutch DIALECT-1 Cohort." KI Reports **4**(5): 710-719.

Ortega, N., C. Carmeli, O. Efthimiou, J. H. Beer, A. V. Gunten, M. Preisig, L. Zullo, J. Vaucher, P. Vollenweider, P. Marques-Vidal, N. Rodondi, A. Chiolero and P. O. Chocano-Bedoya (2024). "Effect of dairy consumption on cognition in older adults: A population-based cohort study." Journal of Nutrition, Health & Aging **28**(2): 100031.

Patterson, R. E., A. R. Kristal, L. F. Tinker, R. A. Carter, M. P. Bolton and T. Agurs-Collins (1999). "Measurement characteristics of the Women's Health Initiative food frequency questionnaire." Ann Epidemiol **9**(3): 178-187.

Pertiwi, K., A. J. Wanders, M. C. Harbers, L. K. Küpers, S. S. Soedamah-Muthu, J. de Goede, P. L. Zock and J. M. Geleijnse (2020). "Plasma and Dietary Linoleic Acid and 3-Year Risk of Type 2 Diabetes After Myocardial Infarction: A Prospective Analysis in the Alpha Omega Cohort." Diabetes Care **43**(2): 358-365.

Pokharel, P., A. Olsen, C. Kyro, A. Tjonneland, K. Murray, L. C. Blekkenhorst, M. U. Jakobsen, C. C. Dahm, C. P. Bondonno, J. M. Hodgson and N. P. Bondonno (2025). "Substituting Potatoes with Other Food Groups and Type 2 Diabetes Risk: Findings from the Diet, Cancer, and Health Study." Journal of Nutrition **155**: 270-279.

Praagman, J., A. P. J. Adolphs, C. T. M. van Rossum, I. Sluijs, Y. T. van der Schouw and J. W. J. Beulens (2016). "Reproducibility and relative validity of a FFQ to estimate the intake of fatty acids." British Journal of Nutrition **115**(12): 2154-2161.

Rimm, E. B., E. L. Giovannucci, M. J. Stampfer, G. A. Colditz, L. B. Litin and W. C. Willett (1992). "Reproducibility and validity of an expanded self-administered semiquantitative food frequency questionnaire among male health professionals." Am J Epidemiol **135**(10): 1114-1126; discussion 1127-1136.

Rivera-Paredez, B., G. León-Reyes, D. Rangel-Marín, J. Salmerón and R. Velázquez-Cruz (2023). "Associations between Macronutrients Intake and Bone Mineral Density: A Longitudinal Analysis of the Health Workers Cohort Study Participants." Journal of Nutrition, Health & Aging **27**(12): 1196-1205.

Salvini, S., D. J. Hunter, L. Sampson, M. J. Stampfer, G. A. Colditz, B. Rosner and W. C. Willett (1989). "Food-based validation of a dietary questionnaire: the effects of week-to-week variation in food consumption." Int J Epidemiol **18**(4): 858-867.

Santiago, S., I. Zazpe, A. Gea, J. M. Nuñez-Córdoba, S. Carlos, M. Bes-Rastrollo and M. A. Martínez-González (2018). "Fat Quality Index and Risk of Cardiovascular Disease in the Sun Project." Journal of Nutrition, Health & Aging **22**(4): 526-533.

Sasanfar, B., F. Toorang, K. Zendehdel and A. Salehi-Abargouei (2022). "Substitution of dietary macronutrients and their sources in association with breast cancer: results from a large-scale case–control study." European Journal of Nutrition **61**(5): 2687-2695.

Scheffers, F. R., J. M. Boer, A. H. Wijga, Y. T. van der Schouw, H. A. Smit and W. M. Verschuren (2022). "Substitution of pure fruit juice for fruit and sugar-sweetened beverages and cardiometabolic risk in European Prospective Investigation into Cancer and Nutrition (EPIC)-NL: a prospective cohort study." Public Health Nutr **25**(6): 1504-1514.

Schmid, D., M. Song, X. Zhang, W. C. Willett, R. Vaidya, E. L. Giovannucci and K. B. Michels (2020). "Yogurt consumption in relation to mortality from cardiovascular disease, cancer, and all causes: a prospective investigation in 2 cohorts of US women and men." American Journal of Clinical Nutrition **111**(3): 689-697.

Seah, J. Y. H., W. P. Koh, J. M. Yuan and R. M. van Dam (2019). "Rice intake and risk of type 2 diabetes: the Singapore Chinese Health Study." European Journal of Nutrition **58**(8): 3349-3360.

Siebelink, E., A. Geelen and J. H. M. de Vries (2011). "Self-reported energy intake by FFQ compared with actual energy intake to maintain body weight in 516 adults." British Journal of Nutrition **106**(2): 274-281.

Slurink, I. A. L., N. R. den Braver, F. Rutters, N. Kupper, T. Smeets, P. J. M. Elders, J. W. J. Beulens and S. S. Soedamah-Muthu (2022). "Dairy product consumption and incident prediabetes in Dutch middle-aged adults: the Hoorn Studies prospective cohort." European Journal of Nutrition **61**(1): 183-196.

Solvoll, K. (1983). Comparison of Dietary Data from Self-Administered Questionnaire and 24 hour Recall [In Norwegian], The Cardiovascular Disease Study in Norwegian Counties. Oslo, Section for Dietary Research, University of Oslo.

Song, M., T. T. Fung, F. B. Hu, W. C. Willett, V. D. Longo, A. T. Chan and E. L. Giovannucci (2016). "Association of Animal and Plant Protein Intake With All-Cause and Cause-Specific Mortality." JAMA Intern Med **176**(10): 1453-1463.

Stiegler, P., S. Sausenthaler, A. E. Buyken, P. Rzehak, D. Czech, J. Linseisen, A. Kroke, K. Gedrich, C. Robertson and J. Heinrich (2010). "A new FFQ designed to measure the intake of fatty acids and antioxidants in children." Public Health Nutr **13**(1): 38-46.

Streppel, M. T., J. H. M. de Vries, S. Meijboom, M. Beekman, A. J. M. de Craen, P. E. Slagboom and E. J. M. Feskens (2013). "Relative validity of the food frequency questionnaire used to assess dietary intake in the Leiden Longevity Study." Nutrition Journal **12**(1): 75.

Stuber, J. M., L. E. T. Vissers, W. M. M. Verschuren, J. M. A. Boer, Y. T. van der Schouw and I. Sluijs (2021). "Substitution among milk and yogurt products and the risk of incident type 2 diabetes in the EPIC-NL cohort." J Hum Nutr Diet **34**(1): 54-63.

Su, Y., B. B. Cochrane, K. Reding, J. R. Herting, L. F. Tinker and O. Zaslavsky (2022). "Mediterranean Diet and Fatigue among Community-Dwelling Postmenopausal Women." J Nutr Gerontol Geriatr **41**(1): 22-45.

Subar, A. F., F. E. Thompson, V. Kipnis, D. Midthune, P. Hurwitz, S. McNutt, A. McIntosh and S. Rosenfeld (2001). "Comparative validation of the Block, Willett, and National Cancer Institute food frequency questionnaires : the Eating at America's Table Study." Am J Epidemiol **154**(12): 1089-1099.

Suga, H., K. Asakura, S. Sasaki, M. Nojima, H. Okubo, N. Hirota, A. Notsu, M. Fukui and C. Date (2018). "Validation study of a self-administered diet history questionnaire for estimating amino acid intake among Japanese adults." Asia Pacific Journal of Clinical Nutrition **27**(3): 638-645.

Sun, C., W. S. Zhang, C. Q. Jiang, Y. L. Jin, X. Q. Deng, G. N. Thomas, J. Woo, K. K. Cheng, T. H. Lam and L. Xu (2023). "Cereal intake and mortality in older Chinese: a 15-year follow-up of a prospective cohort study." European Journal of Nutrition **62**(3): 1239-1251.

Takahashi, K., Y. Yoshimura, T. Kaimoto, D. Kunii, T. Komatsu and S. Yamamoto (2001). "Validation of a Food Frequency Questionnaire Based on Food Groups for Estimating Individual Nutrient Intake." The Japanese Journal of Nutrition and Dietetics **59**(5): 221-232.

Tessier, A. J., M. Cortese, C. Yuan, K. Bjornevik, A. Ascherio, D. D. Wang, J. E. Chavarro, M. J. Stampfer, F. B. Hu, W. C. Willett and M. Guasch-Ferre (2024). "Consumption of Olive Oil and Diet Quality and Risk of Dementia-Related Death." JAMA Network Open **7**(5): e2410021.

Thao, U., M. Lajous, N. Laouali, G. Severi, M.-C. Boutron-Ruault and C. J. MacDonald (2023). "Relative to processed red meat, alternative protein sources are associated with a lower risk of hypertension and diabetes in a prospective cohort of French women." British Journal of Nutrition **129**(11): 1964-1975.

Thompson, F. E., V. Kipnis, D. Midthune, L. S. Freedman, R. J. Carroll, A. F. Subar, C. C. Brown, M. S. Butcher, T. Mouw, M. Leitzmann and A. Schatzkin (2008). "Performance of a food-frequency questionnaire in the US NIH–AARP (National Institutes of Health–American Association of Retired Persons) Diet and Health Study." Public Health Nutrition **11**(2): 183-195.

Tjønneland, A., K. Overvad, J. Haraldsdóttir, S. Bang, M. Ewertz and O. M. Jensen (1991). "Validation of a semiquantitative food frequency questionnaire developed in Denmark." Int J Epidemiol **20**(4): 906-912.

Tooze, J. A., N. S. The, J. L. Crandell, S. C. Couch, E. J. Mayer-Davis, C. Koebnick and A. D. Liese (2020). "An Approach for Examining the Impact of Food Group-Based Sources of Nutrients on Outcomes with Application to PUFAs and LDL in Youth with Type 1 Diabetes." Nutrients **12**(4): 941.

van Eekelen, E., J. W. J. Beulens, A. Geelen, V. B. Schrauwen-Hinderling, H. Lamb, A. de Roos, F. Rosendaal and R. de Mutsert (2019). "Consumption of Alcoholic and Sugar-Sweetened Beverages is Associated with Increased Liver Fat Content in Middle-Aged Men and Women." J Nutr **149**(4): 649-658.

van Liere, M. J., F. Lucas, F. Clavel, N. Slimani and S. Villeminot (1997). "Relative validity and reproducibility of a French dietary history questionnaire." International Journal of Epidemiology **26**(suppl_1): S128-S128.

Venø, S. K., C. S. Bork, M. U. Jakobsen, S. Lundbye-Christensen, F. W. Bach, P. L. McLennan, A. Tjønneland, E. B. Schmidt and K. Overvad (2018). "Substitution of Fish for Red Meat or Poultry and Risk of Ischemic Stroke." Nutrients **10**(11): 1648.

Verkleij-Hagoort, A. C., J. H. M. de Vries, M. P. G. Stegers, J. Lindemans, N. T. C. Ursem and R. P. M. Steegers-Theunissen (2007). "Validation of the assessment of folate and vitamin B12 intake in women of reproductive age: the method of triads." European Journal of Clinical Nutrition **61**(5): 610-615.

Verspoor, E., T. Voortman, F. J. A. van Rooij, F. Rivadeneira, O. H. Franco, J. C. Kiefte-de Jong and J. D. Schoufour (2020). "Macronutrient intake and frailty: the Rotterdam Study." European Journal of Nutrition **59**(7): 2919-2928.

Vissers, L. E. T., J. Rijksen, J. M. A. Boer, W. M. M. Verschuren, Y. T. van der Schouw and I. Sluijs (2019). "Fatty acids from dairy and meat and their association with risk of coronary heart disease." Eur J Nutr **58**(7): 2639-2647.

Voortman, T., Z. Chen, C. Girschik, M. Kavousi, O. H. Franco and K. V. E. Braun (2021). "Associations between macronutrient intake and coronary heart disease (CHD): The Rotterdam Study." Clin Nutr **40**(11): 5494-5499.

Würtz, A. M. L., M. D. Hansen, A. Tjønneland, E. B. Rimm, E. B. Schmidt, K. Overvad and M. U. Jakobsen (2021a). "Replacement of potatoes with other vegetables and risk of myocardial infarction in the Danish Diet, Cancer and Health cohort." Br J Nutr **126**(11): 1709-1716.

Würtz, A. M. L., M. U. Jakobsen, M. L. Bertoia, T. Hou, E. B. Schmidt, W. C. Willett, K. Overvad, Q. Sun, J. E. Manson, F. B. Hu and E. B. Rimm (2021b). "Replacing the consumption of red meat with other major dietary protein sources and risk of type 2 diabetes mellitus: a prospective cohort study." Am J Clin Nutr **113**(3): 612-621.

Wan, Y., K. Wu, L. Wang, K. Yin, M. Song, E. L. Giovannucci and W. C. Willett (2022). "Dietary fat and fatty acids in relation to risk of colorectal cancer." European Journal of Nutrition **61**(4): 1863-1873.

Wang, M., H. Ma, Q. Song, T. Zhou, Y. Hu, Y. Heianza, J. E. Manson and L. Qi (2022). "Red meat consumption and all-cause and cardiovascular mortality: results from the UK Biobank study." Eur J Nutr **61**(5): 2543-2553.

Watanabe, D., K. Maruyama, A. Tamakoshi, I. Muraki and Jacc Study Group (2024). "Association between Diet-Related Greenhouse Gas Emissions and Mortality among Japanese Adults: The Japan Collaborative Cohort Study." Environmental Health Perspectives **132**(11): 117002-117001-117002-117010.

Wesselink, A. K., S. K. Willis, A. S. D. Laursen, E. M. Mikkelsen, T. R. Wang, E. Trolle, K. L. Tucker, K. J. Rothman, L. A. Wise and E. E. Hatch (2022). "Protein-rich food intake and risk of spontaneous abortion: a prospective cohort study." Eur J Nutr **61**(5): 2737-2748.

Willett, W. C., L. Sampson, M. J. Stampfer, B. Rosner, C. Bain, J. Witschi, C. H. Hennekens and F. E. Speizer (1985). "Reproducibility and validity of a semiquantitative food frequency questionnaire." Am J Epidemiol **122**(1): 51-65.

Woo, J., S. S. F. Leung, S. C. Ho, T. H. Lam and E. D. Janus (1997). "A food frequency questionnaire for use in the Chinese population in Hong Kong: Description and examination of validity." Nutrition Research **17**(11): 1633-1641.

Wu, F., B. Wang, P. Zhuang, Z. Lu, Y. Li, H. Wang, X. Liu, X. Zhao, W. Yang, J. Jiao, W. Zheng and Y. Zhang (2022). "Association of preserved vegetable consumption and prevalence of colorectal polyps: results from the Lanxi Pre-colorectal Cancer Cohort (LP3C)." European Journal of Nutrition **61**(3): 1273-1284.

Yang, J., Q. Chang, X. Tian, B. Zhang, L. Zeng, H. Yan, S. Dang and Y.-H. Li (2022a). "Dietary protein intake during pregnancy and birth weight among Chinese pregnant women with low intake of protein." Nutrition & Metabolism **19**(1): 43.

Yang, J., D. K. Tobias, S. Li, S. N. Bhupathiraju, S. H. Ley, S. N. Hinkle, F. Qian, Z. Chen, Y. Zhu, W. Bao, J. E. Chavarro, F. B. Hu and C. Zhang (2022b). "Habitual coffee consumption and subsequent risk of type 2

diabetes in individuals with a history of gestational diabetes – a prospective study." American Journal of Clinical Nutrition **116**(6): 1693-1703.

Ying, A. F., M. Talaei, D. J. Hausenloy and W. P. Koh (2024). "Consumption of different types of meat and the risk of chronic limb-threatening ischemia: the Singapore Chinese Health Study." Nutrition Journal **23**(1): 103.

Yokoyama, Y., R. Takachi, J. Ishihara, Y. Ishii, S. Sasazuki, N. Sawada, Y. Shinozawa, J. Tanaka, E. Kato, K. Kitamura, K. Nakamura and S. Tsugane (2016). "Validity of Short and Long Self-Administered Food Frequency Questionnaires in Ranking Dietary Intake in Middle-Aged and Elderly Japanese in the Japan Public Health Center-Based Prospective Study for the Next Generation (JPHC-NEXT) Protocol Area." J Epidemiol **26**(8): 420-432.

Yoshioka, M., K. Kosaki, M. Matsui, S. Mori, N. Nishitani, C. Saito, K. Yamagata, O. M. Kuro and S. Maeda (2023). "Association between the intake of plant and animal proteins and the serum fibroblast growth factor-23 level in patients with chronic kidney disease analyzed by the isocaloric substitution model." Endocr J **70**(1): 31-42.

Yuan, C., D. Spiegelman, E. B. Rimm, B. A. Rosner, M. J. Stampfer, J. B. Barnett, J. E. Chavarro, J. C. Rood, L. J. Harnack, L. K. Sampson and W. C. Willett (2018). "Relative Validity of Nutrient Intakes Assessed by Questionnaire, 24-Hour Recalls, and Diet Records as Compared With Urinary Recovery and Plasma Concentration Biomarkers: Findings for Women." Am J Epidemiol **187**(5): 1051-1063.

Yuan, C., D. Spiegelman, E. B. Rimm, B. A. Rosner, M. J. Stampfer, J. B. Barnett, J. E. Chavarro, A. F. Subar, L. K. Sampson and W. C. Willett (2017). "Validity of a Dietary Questionnaire Assessed by Comparison With Multiple Weighed Dietary Records or 24-Hour Recalls." American Journal of Epidemiology **185**(7): 570-584.

Yuzbashian, E., G. Asghari, P. Mirmiran, C. B. Chan and F. Azizi (2021a). "Changes in dairy product consumption and subsequent type 2 diabetes among individuals with prediabetes: Tehran Lipid and Glucose Study." Nutrition Journal **20**(1): 88.

Yuzbashian, E., M. Nosrati-Oskouie, G. Asghari, C. B. Chan, P. Mirmiran and F. Azizi (2021b). "Associations of dairy intake with risk of incident metabolic syndrome in children and adolescents: Tehran Lipid and Glucose Study." Acta Diabetologica **58**(4): 447-457.

Zhao, B., L. Gan, B. I. Graubard, S. Mannisto, F. Fang, S. J. Weinstein, L. M. Liao, R. Sinha, X. Chen, D. Albanes and J. Huang (2024). "Plant and Animal Fat Intake and Overall and Cardiovascular Disease Mortality." JAMA Internal Medicine **184**(10): 1234-1245.

Zhu, Y., M. M. Hedderson, S. Sridhar, F. Xu, J. Feng and A. Ferrara (2019). "Poor diet quality in pregnancy is associated with increased risk of excess fetal growth: a prospective multi-racial/ethnic cohort study." International Journal of Epidemiology **48**(2): 423-432.

Zimorovat, A., F. Moghtaderi, M. Amiri, H. Raeisi-Dehkordi, M. Mohyadini, M. Mohammadi, S. Zarei, E. Karimi-Nazari, M. Mirzaei, A. Nadjarzadeh and A. Salehi-Abargouei (2022). "Validity and Reproducibility of a Semiquantitative Multiple-Choice Food Frequency Questionnaire in Iranian Adults." Food Nutr Bull **43**(2): 171-188.
